# Unveiling the Phytochemical Diversity and Bioactivity of *Astragalus melanophrurius*: A First Report Integrating Experimental and In Silico Approaches

**DOI:** 10.3390/ph18010103

**Published:** 2025-01-15

**Authors:** Gulcan Gencer, Cengiz Sarikurkcu, Bektas Tepe

**Affiliations:** 1 Department of Biostatistics and Medical Informatics, Faculty of Medicine, Afyonkarahisar Health Sciences University, 03030 Afyonkarahisar, Türkiye; 2 Department of Analytical Chemistry, Faculty of Pharmacy, Afyonkarahisar Health Sciences University, 03100 Afyonkarahisar, Türkiye; sarikurkcu@gmail.com; 3 Department of Molecular Biology and Genetics, Faculty of Science, Kilis 7 Aralik University, 79000 Kilis, Türkiye; bektastepe@kilis.edu.tr

**Keywords:** *Astragalus* species, secondary metabolites, LC–MS phenolic analysis, free radical scavenging, cholinesterase inhibition, in silico interactions

## Abstract

**Background**: The genus *Astragalus* is renowned for its diverse bioactive potential, yet the chemical composition and biological properties of *Astragalus melanophrurius* remain inadequately explored. This study aimed to investigate the chemical profile, antioxidant capacity, and enzyme inhibitory activities of methanol extracts from various plant parts of *A. melanophrurius*. **Methods**: Methanol extracts were obtained from leaves, stems, flowers, roots, and aerial portions of *A. melanophrurius*. The chemical composition was determined using LC–ESI–MS/MS, focusing on key phytochemicals such as hyperoside, kaempferol, 4-hydroxybenzoic acid, and chlorogenic acid. Antioxidant activities were assessed via DPPH, ABTS, and FRAP assays, while enzyme inhibitory activities were evaluated against α-amylase and tyrosinase. In silico molecular docking analyses were conducted to explore the interactions between major compounds and target enzymes. **Results**: The leaf extract exhibited the highest total phenolic and flavonoid contents, correlating with superior antioxidant activities, achieving IC_50_ values of 16.55 mg/mL, 4.58 mg/mL, and 3.07 mg/mL in DPPH, ABTS, and FRAP assays, respectively. The root extract demonstrated notable α-amylase (IC_50_ = 2.99 mg/mL) and tyrosinase (IC_50_ = 1.34 mg/mL) inhibitory activities, suggesting potential applications in diabetes and hyperpigmentation management. Molecular docking revealed stable complexes of hyperoside and kaempferol with target enzymes, supporting their roles in observed bioactivities. **Conclusions**: This study highlights the bioactivity of *A. melanophrurius* extracts, particularly from leaves and roots, supporting their therapeutic potential. Future research should focus on isolating active compounds and conducting in vivo studies to confirm efficacy and elucidate mechanisms of action.

## 1. Introduction

Plant-based medicines have gained significant attention as therapeutic options for various health conditions due to limitations and side effects associated with synthetic drugs. Unlike synthetic compounds, phytochemicals—secondary metabolites derived from plants—offer diverse pharmacological activities with a higher margin of safety [1,2]. The growing interest in herbal remedies is particularly linked to advancements in understanding the pathways of diseases such as diabetes and oxidative stress-related disorders. Oxidative stress, which results from an imbalance between reactive oxygen species (ROS) production and the body’s antioxidant defense systems, has driven research into natural antioxidants with pharmacological potential [3,4,5].

ROS-induced oxidative stress is associated with chronic inflammatory and proliferative diseases [6]. Synthetic antioxidants, such as butylated hydroxyanisole (BHA), have been employed to mitigate oxidative stress and inhibit ROS production. However, concerns about their potential carcinogenic risks, as highlighted by studies on genotoxic effects [7,8], have prompted a shift toward natural alternatives with safer profiles [6,9,10].

Diabetes, characterized by dysfunction of pancreatic β cells and impaired glucose and lipid metabolism, is a major global health issue, with type II diabetes accounting for nearly 90% of cases. If left untreated, it can result in serious complications, including organ failure [11]. Significant advancements in diabetes management have focused on controlling postprandial hyperglycemia through the inhibition of enzymes involved in carbohydrate digestion—specifically α-amylase and α-glucosidase [12,13,14,15]. While synthetic hypoglycemic agents like metformin are widely used, their long-term safety and efficacy remain concerns, driving the search for natural enzyme inhibitors derived from plants [16,17,18].

Melanin plays an essential role in shielding human skin from ultraviolet radiation. However, excessive melanin production can result in hyperpigmentation disorders, neurodegenerative diseases, and even skin cancer [19,20,21]. Tyrosinase inhibitors, which modulate melanin synthesis, have emerged as therapeutic agents for managing hyperpigmentation and preserving fruits by preventing browning [22,23,24,25]. Despite their effectiveness, many synthetic tyrosinase inhibitors pose safety and efficacy concerns, leading to increased interest in plant-derived alternatives with fewer adverse effects [26,27,28].

The genus *Astragalus*, belonging to the Fabaceae family, represents the largest group of angiosperms, comprising approximately 3000 species as per The Plant List. This genus has been integral to traditional medicine for over two millennia, particularly in Chinese folk medicine, addressing conditions such as hypertension, chronic bronchitis, stomach ulcers, diabetes, and gynecological disorders [29,30,31,32,33]. The bioactive compounds in Astragalus extracts exhibit a wide spectrum of pharmacological effects, including anti-inflammatory, immunostimulatory, anticancer, anti-diabetic, and cardioprotective activities [34,35,36,37,38,39].

In recent years, computational approaches like molecular docking have become indispensable tools in phytochemical research. Molecular docking facilitates the exploration of binding interactions between small molecules and target proteins, offering insights into the contributions of individual phytochemicals to observed biological effects [40,41,42,43]. This computational method complements experimental studies by predicting binding affinities and interaction mechanisms, thereby enhancing our understanding of plant-derived compounds’ therapeutic potential.

The endemic species *Astragalus melanophrurius* Boiss. has remained largely unexplored in terms of its phytochemical composition and biological activities. Given its potential therapeutic properties, this species represents a promising candidate for detailed investigation. While extensive research has been conducted on other *Astragalus* species, no prior studies have specifically addressed the antioxidant and enzyme inhibitory properties of *A. melanophrurius*. To address this gap, the present study evaluates methanolic extracts from the plant’s leaves, stems, flowers, roots, and aerial parts. The phytochemical profiles were analyzed both qualitatively and quantitatively, and antioxidant activities were assessed using various assays, including ABTS and DPPH radical scavenging, phosphomolybdenum total antioxidant capacity, ferrous ion chelation, and reducing power (CUPRAC/FRAP). Additionally, enzyme inhibitory activities against α-amylase and tyrosinase were systematically evaluated.

To better understand the bioactive potential of *A. melanophrurius*, the study further explores the correlations between major phytochemicals identified in the extracts and their corresponding bioactivities through statistical analyses. In parallel, molecular docking simulations were performed to examine the binding interactions of key compounds with α-amylase and tyrosinase—two enzymes implicated in conditions such as diabetes and skin pigmentation disorders. This integrative approach sheds light on the potential health benefits of *A. melanophrurius*, providing novel insights into its phytopharmacological properties. By filling a significant gap in the literature, this study contributes valuable data that may support future pharmacological applications and inspire further research on underexplored endemic plant species.

## 2. Results

### 2.1. Chemical Composition

**Figure 1:**
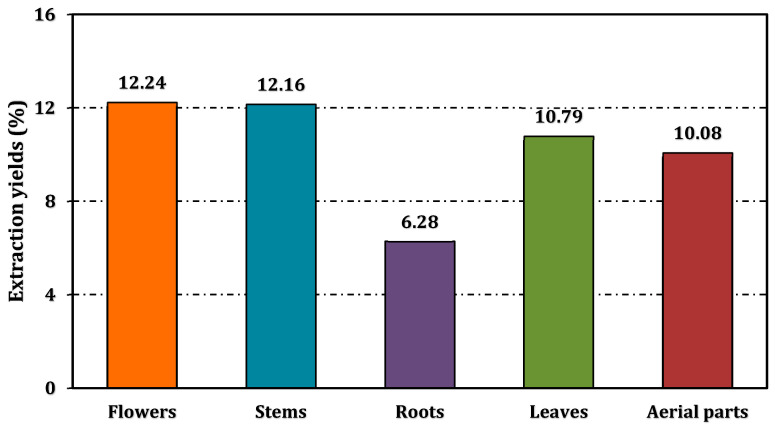
Extraction yields of methanol extracts from *A. melanophrurius*, expressed as the percentage of dried plant material extracted into methanol.

The extraction yields of *A. melanophrurius* methanol extracts exhibited significant variability among plant parts, calculated as the percentage of dried plant material extracted into methanol. The flower extract achieved the highest yield (12.24%), followed closely by the stem (12.16%) and leaf extracts (10.79%). The aerial portion and root extracts yielded 10.08% and 6.28%, respectively (Figure 1).

**Figure 2:**
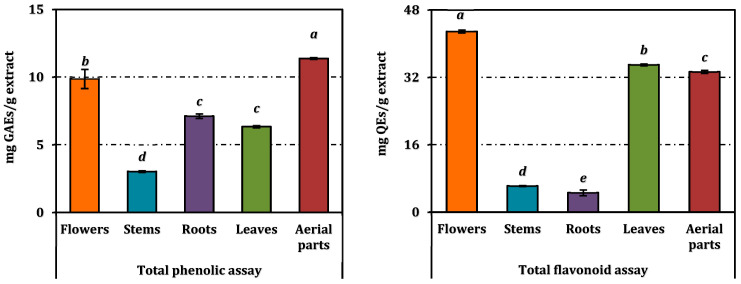
Total flavonoid and phenolic contents of *A. melanophrurius* extracts. REs and QEs: Rutin and quercetin equivalents, respectively. Values indicated by the same superscripts are not different from the honestly significant difference after Tukey’s hoc test at 5% significance level.

The aerial part extract displayed the highest total phenolic content (TPC) at 11.37 mg GAEs/g extract, significantly surpassing all other extracts (*p* < 0.05). The flower extract (9.87 mg GAEs/g) ranked second, while the root (7.12 mg GAEs/g) and leaf extracts (6.34 mg GAEs/g) exhibited comparable TPC levels, significantly higher than the stem extract (3.03 mg GAEs/g) (Figure 2).

Regarding total flavonoid content (TFC), the flower extract demonstrated the most abundant concentration (42.9 mg QEs/g extract), followed by the leaf (35.0 mg QEs/g) and aerial part extracts (33.31 mg QEs/g). The stem and root extracts contained significantly lower TFC values (6.22 and 4.61 mg QEs/g, respectively). These results align with the high extract yields and phenolic richness observed in the aerial and flower extracts, underscoring their bioactive potential (Figure 2).

The LC–ESI–MS/MS analysis revealed distinct chemical profiles across the extracts (Table 1). Hyperoside was highly concentrated in the leaf extract (573.21 µg/g), significantly exceeding its levels in the aerial part (242.19 µg/g), stem (16.68 µg/g) and flower extracts (14.65 µg/g). Similarly, 4-hydroxybenzoic acid showed its highest concentration in the flower extract (202.74 µg/g), with comparatively moderate levels in the aerial part (80.56 µg/g) and root extracts (38.12 µg/g). Notably, kaempferol levels peaked in the flower extract (63.44 µg/g), surpassing its concentration in aerial parts (34.19 µg/g) and leaves (10.05 µg/g).

Certain compounds, such as chlorogenic acid (28.77 µg/g), p-coumaric acid (26.19 µg/g), luteolin 7-glucoside (21.48 µg/g), ferulic acid (21.04 µg/g), and verbascoside (12.23 µg/g), were most abundant in the aerial part extract, corroborating its superior TPC and TFC values. In contrast, specific phenolics like protocatechuic acid (15.01 µg/g) and rosmarinic acid (14.09 µg/g) were particularly enriched in leaf extracts.

### 2.2. Antioxidant Activity

**Figure 3:**
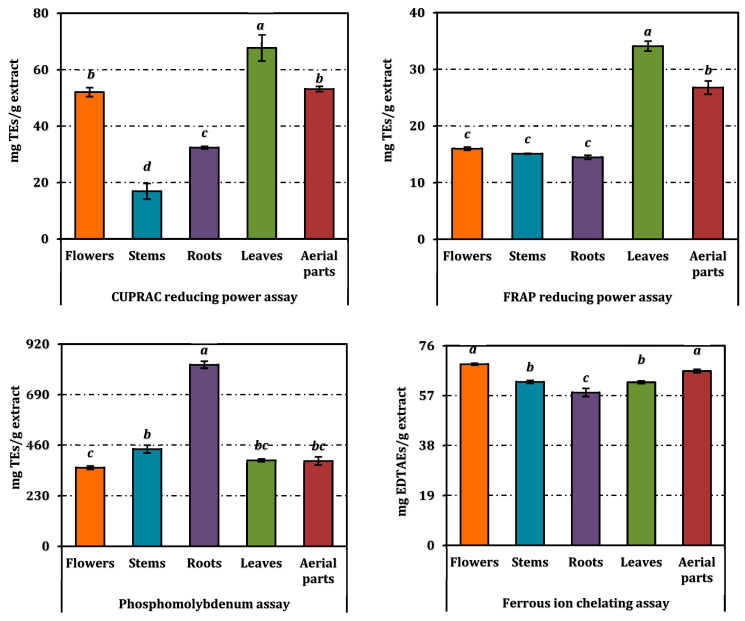

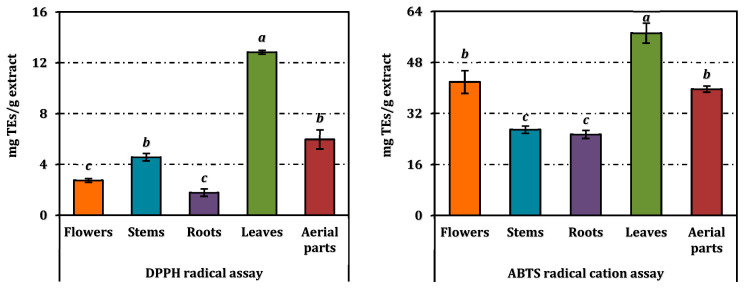
Antioxidant activity of *A. melanophrurius* extracts. Values indicated by the same superscripts are not different from the honestly significant difference after Tukey’s hoc test at 5% significance level.

**Figure 4:**
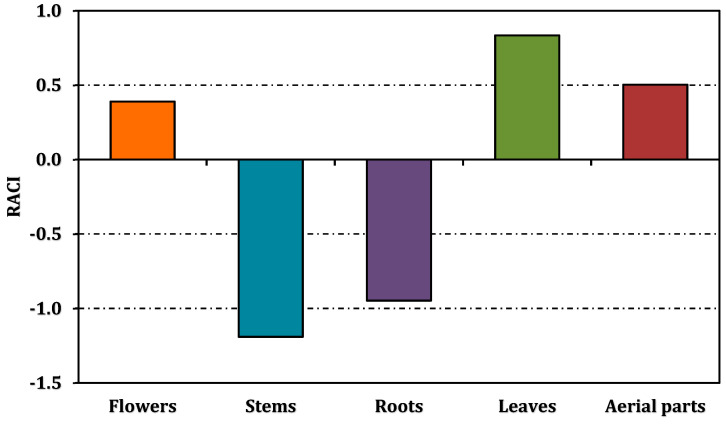
Relative antioxidant capacity index of *A. melanophrurius* extracts.

The antioxidant activities of the methanolic extracts from different parts of *A. melanophrurius* were assessed using six assays, each designed to explore distinct mechanisms of antioxidant action (Table 2, Figure 3). These assays included DPPH radical scavenging, ABTS radical cation scavenging, ferrous ion chelation, phosphomolybdenum, CUPRAC, and FRAP reducing power. Additionally, the RACI values enabled a comprehensive comparison of the extracts’ overall antioxidant capacities (Figure 4).

The leaf extract exhibited the strongest DPPH scavenging activity (IC_50_ = 16.55 mg/mL), followed by the stem extract (IC_50_ = 35.32 mg/mL) and flower extract (IC_50_ = 47.08 mg/mL). Both the aerial parts and root extracts demonstrated weak activity, with IC_50_ values exceeding 20 mg/mL. The low IC_50_ value of the leaf extract indicates a greater efficiency in neutralizing free radicals, potentially attributed to its higher phenolic and flavonoid content. The leaf extract also excelled in the ABTS assay (IC_50_ = 4.58 mg/mL), significantly outperforming other extracts. The flower extract (IC_50_ = 6.19 mg/mL) and aerial parts extract (IC_50_ = 6.50 mg/mL) displayed moderate activity, while the root (IC_50_ = 9.83 mg/mL) and stem (IC_50_ = 9.32 mg/mL) extracts were less effective. The ABTS results suggest that the leaf extract is particularly effective in scavenging both polar and nonpolar radicals.

Ferrous ion chelation revealed less variability among extracts. The flower extract displayed the strongest chelating activity (IC_50_ = 1.04 mg/mL), followed by the aerial parts (IC_50_ = 1.09 mg/mL), stem, leaf (IC_50_ = 1.16 mg/mL), and root extracts (IC_50_ = 1.24 mg/mL). Despite their similar performance, the flower extract’s slightly superior chelating ability may be linked to specific secondary metabolites conducive to metal ion binding.

The root extract was the most potent in the phosphomolybdenum assay (EC_50_ = 1.40 mg/mL), while the flower, leaf, and aerial parts extracts showed comparable, moderate activity (EC_50_ values of 3.23, 2.95, and 2.98 mg/mL, respectively). The root’s higher reducing power suggests a greater concentration of compounds capable of electron donation, such as phenolic acids or flavonoids.

In the CUPRAC assay, the leaf extract again displayed the highest activity (EC_50_ = 4.12 mg/mL), followed by the aerial parts (EC_50_ = 5.03 mg/mL) and flower extracts (EC_50_ = 5.12 mg/mL). The root and stem extracts were less effective, with EC_50_ values of 7.35 and 11.29 mg/mL, respectively. This indicates that the leaf extract is particularly efficient in reducing copper ions. The leaf extract achieved the lowest EC_50_ value (3.07 mg/mL) in the FRAP assay, highlighting its superior ferric ion-reducing ability. The aerial parts extract (EC_50_ = 3.91 mg/mL) followed, with the flower (6.54 mg/mL), stem (6.92 mg/mL), and root extracts (7.22 mg/mL) showing less pronounced activity.

The RACI values synthesize the data from all assays, enabling a holistic ranking of the extracts’ antioxidant activities. The leaf extract demonstrated the highest RACI value (0.83), affirming its superior antioxidant potential. The aerial parts (0.50) and flower extracts (0.39) followed, while the root (−0.95) and stem extracts (−1.19) were less active (Figure 4).

### 2.3. Enzyme Inhibitory Activity

**Figure 5:**
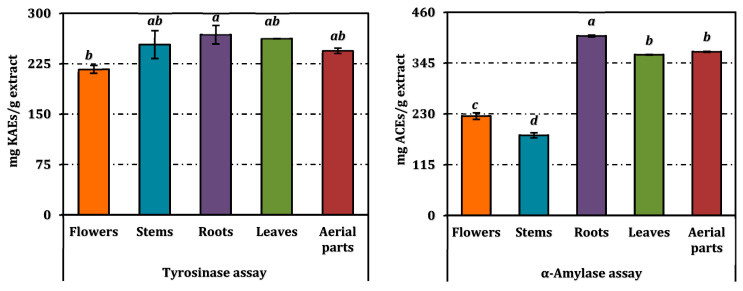
Enzyme inhibition activity of *A. melanophrurius* extracts. Values indicated by the same superscripts are not different from the honestly significant difference after Tukey’s hoc test at 5% significance level.

The enzyme inhibitory potential of methanolic extracts derived from different parts of *A. melanophrurius* was assessed against tyrosinase and α-amylase, two enzymes with significant pharmacological relevance (Table 3, Figure 5). The results, presented as IC_50_ values in Table 3, demonstrate variations in the inhibitory activities among the extracts, reflecting differences in their phytochemical compositions.

Tyrosinase is a key enzyme in melanogenesis, and its inhibition is relevant in the treatment of hyperpigmentation disorders. Among the tested extracts, the root extract exhibited the strongest tyrosinase inhibition (IC_50_ = 1.34 mg/mL), followed closely by the leaf extract (IC_50_ = 1.37 mg/mL) and aerial part extract (IC_50_ = 1.47 mg/mL). The stem extract (IC_50_ = 1.42 mg/mL) and flower extract (IC_50_ = 1.65 mg/mL) showed slightly lower activities but were statistically distinct from the root and leaf extracts at a 5% significance level. The positive control, kojic acid, displayed an IC_50_ of 0.37 mg/mL, indicating that the extracts possess moderate inhibitory potential compared to this standard compound. The high activity of the root extract may be attributed to its enrichment in phenolics and flavonoids, which are known tyrosinase inhibitors. Similarly, the leaf extract’s strong activity aligns with its substantial phenolic content, as observed in the compositional analysis.

α-amylase inhibition is a key mechanism in managing postprandial hyperglycemia in diabetic patients. The root extract again emerged as the most potent inhibitor (IC_50_ = 2.99 mg/mL), followed by the aerial part (IC_50_ = 3.28 mg/mL) and leaf (IC_50_ = 3.34 mg/mL) extracts, with no significant differences among these three. The flower extract demonstrated relatively weaker inhibition (IC_50_ = 5.36 mg/mL), and the stem extract showed the least activity (IC_50_ = 6.62 mg/mL). Acarbose, the positive control, exhibited significantly higher activity (IC_50_ = 1.19 mg/mL) compared to all extracts. The strong inhibitory effect of the root extract can be associated with its high levels of bioactive flavonoids, which have been implicated in α-amylase inhibition. The comparable activities of the aerial part and leaf extracts may result from shared phenolic profiles.

### 2.4. Correlations Among Phenolic Compounds and Assays

The correlation analysis between the chemical composition of *A. melanophrurius* extracts and their bioactivities revealed distinct relationships among phenolic compounds, flavonoids, and antioxidant assays (Table 4). Total flavonoid content exhibited strong positive correlations with most antioxidant assays, particularly ABTS (r = 0.799), CUPRAC (r = 0.850), and FICA (r = 0.793), suggesting that flavonoids significantly contribute to radical scavenging and reducing power activities. In contrast, the total phenolic content showed weaker correlations with these assays, such as CUPRAC (r = 0.590) and FICA (r = 0.601), indicating that while phenolics play a role, flavonoids may be the primary contributors to the observed bioactivities.

Among individual phenolic compounds, p-coumaric acid demonstrated the highest correlation with FRAP (r = 0.871), CUPRAC (r = 0.740), and ABTS (r = 0.674), emphasizing its significant role in reducing power and radical scavenging activities. Similarly, hyperoside strongly correlated with FRAP (r = 0.978), ABTS (r = 0.861), and DPPH (r = 0.969), underscoring its potent antioxidant properties across multiple assays. Conversely, ferulic acid showed moderate positive correlations with FRAP (r = 0.425) and DPPH (r = 0.549), but a negative correlation with TAP (r = −0.772), highlighting variability in its activity depending on the assay.

Vanillic acid, despite its known bioactivity, displayed negative correlations with ABTS (r = −0.557) and CUPRAC (r = −0.439), suggesting it may not significantly contribute to these specific antioxidant mechanisms. Interestingly, luteolin 7-glucoside and kaempferol showed relatively weak or inconsistent correlations with most assays, such as CUPRAC (r = 0.270 and 0.432, respectively) and FICA (r = 0.465 and 0.926, respectively), indicating their roles may be context dependent or secondary to other compounds.

These results collectively suggest that the antioxidant potential of *A. melanophrurius* extracts arises from a synergistic interaction between major flavonoids and phenolic acids, with compounds like hyperoside and p-coumaric acid being the most influential contributors to reducing power and radical scavenging activities. However, the variability in correlations across assays highlights the complexity of these interactions and the assay-specific contributions of different phytochemicals.

### 2.5. Analysis of Statistical Analysis Results

The statistical analysis presented in this section aims to provide a comprehensive overview of the relationships between the chemical composition and bioactivity profiles of *A. melanophrurius* extracts discussed in the previous sections. Given the variability observed in the extraction yields, phenolic content, antioxidant activities, and enzyme inhibitory properties, principal component analysis (PCA) offers a valuable approach to visualizing and interpreting the underlying patterns in the dataset. The results of the PCA will help clarify which compounds and bioactivities are most influential in differentiating the plant parts, thereby reinforcing the key findings reported in Section 2.1, Section 2.2, Section 2.3 and Section 2.4.

**Figure 6:**
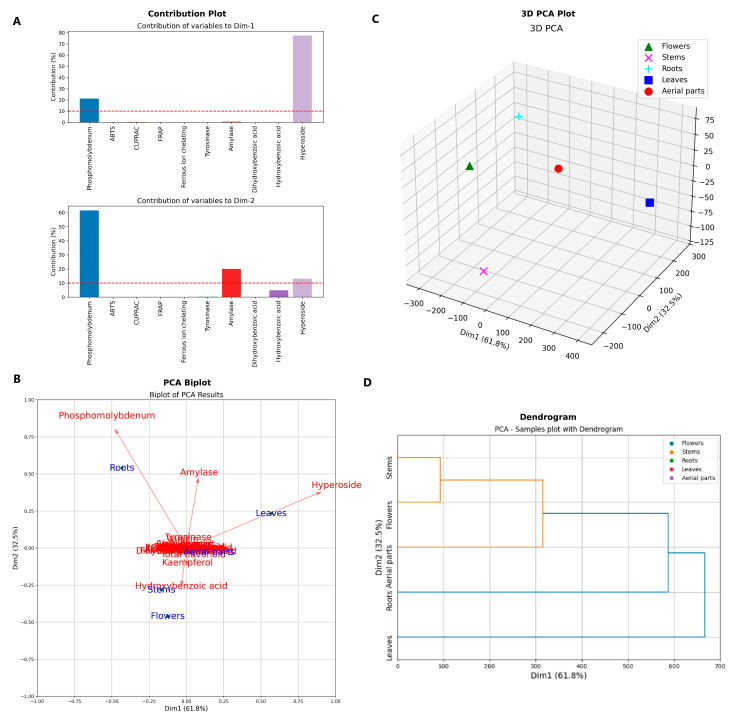
Principle component analysis (PCA) and hierarchical clustering analysis. (**A**) Loading plot. (**B**) Contribution of biological activities to significant dimension of PCA. (**C**) 3D samples plot. (**D**) Hierarchical clustering on the fact.

In this study, principal component analysis (PCA) was conducted to evaluate the relationships among different plant parts based on their antioxidant and enzyme inhibitor activities (Figure 6). In Figure 6A, hyperoside is the variable contributing the most to Dim1, indicating that a significant portion of the variance in the dataset is explained by this variable. Phosphomolybdenum is the second-highest contributor, also playing an important role. The contributions of other variables are quite low, indicating that they have minimal influence on Dim1. Phosphomolybdenum is the variable contributing the most to Dim2 and has a significant impact on both Dim1 and Dim2, suggesting that this variable explains a substantial portion of the variance in the dataset.

In Figure 6B, vectors represent each variable’s contribution to the two principal components. The vectors for phosphomolybdenum and hyperoside are notably long and distinct, suggesting their critical role in explaining the variance in the data. Phosphomolybdenum has a particularly strong influence on Dim1, while hyperoside has significant effects on both Dim1 and Dim2. α-amylase shows a stronger effect on Dim2. Other variables (e.g., tyrosinase, vanillin) are more centrally positioned, indicating they have less influence on these components. The blue dots on the graph (flower, stems, root, leaves, and aerial parts) represent the samples. The leaves are aligned in the same direction as hyperoside, indicating that this organ is significantly influenced by hyperoside. The root is positioned close to phosphomolybdenum, suggesting it is affected by phosphomolybdenum levels.

In Figure 6C, the points on the graph represent different organs, with each organ shown in a distinct color and shape. Roots are positioned higher and farther away from the other organs, indicating significant differences from the rest. Aerial parts have positive values, suggesting high variance in this organ. Leaf parts are positioned close to the aerial parts, indicating some similarities between them.

In Figure 6D, the PCA results are combined with a dendrogram. The graph shows how the organs are distributed along the Dim1 (61.8%) and Dim2 (32.5%) components and grouped through hierarchical clustering. The stems and flower organs are in the first cluster, indicating their chemical compositions are similar. The aerial part forms its own second cluster, suggesting its chemical profile is distinct and unique from the others. The root and leaf organs are in the third cluster, indicating their chemical compositions are similar. The separation of the root, leaf, and aerial parts into different groups highlights the significant differences in their chemical compositions compared to others.

### 2.6. In Silico Analysis

Montbretin A, the co-crystallized inhibitor of human pancreatic α-amylase (AAMY), exhibited a highly favorable binding free energy (ΔG: −9.03 kcal/mol) indicating strong binding affinity for the active site of the enzyme (Table 5). The compound formed a total of 8 classical H bonds with key residues in the active site, including Ile148 (3.01 Å), Tyr151 (2.57 Å), Thr163 (1.97 Å, 2.21 Å, 3.19 Å), His201 (2.07 Å), Glu233 (2.56 Å), and His299 (1.87 Å), highlighting significant polar interactions that stabilize the ligand–receptor complex (Table 5, see Supplementary Figure S1). Additionally, montbretin A formed non-classical H bonds through interactions with Tyr151 (3.49 Å) and His305 (3.72 Å). Montbretin A also showed hydrophobic contacts with Leu162 (5.13 Å), as well as π–lone pair interactions with Tyr62 (2.83 Å), which supported its stabilization within the active site of AAMY (Table 5 and Supplementary Figure S1).

The binding affinity (ΔG) values and intermolecular interactions of AAMY with chlorogenic acid and 4-hydroxybenzoic acid are presented in Table 5 and Supplementary Figures S2 and S3 (see Supplementary Materials). Both chlorogenic acid and 4-hydroxybenzoic acid exhibited energetically weak interactions against the AAMY enzyme, with binding free energy values of −3.80 kcal/mol and −4.12 kcal/mol, respectively (Table 5 and Supplementary Materials).

**Figure 7:**
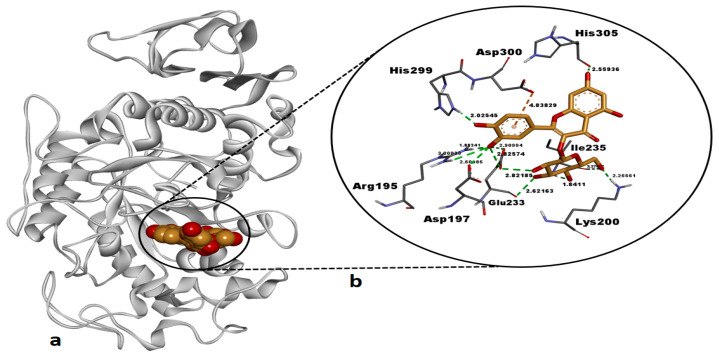
Post-docking top-ranked conformation of hyperoside in complex with the active site of human pancreatic alpha-amylase (AAMY). (**a**) 3D general view of the AAMY–hyperoside complex, with AAMY represented as a solid ribbon model and hyperoside in CPK mode; (**b**) zoomed-in interaction view of hyperoside within the active site of AAMY. Green dashed lines indicate conventional hydrogen bonds, while the orange dashed line represent electrostatic interaction. Non-bonded interaction distances (Å) are displayed in bold black. Images were rendered and prepared using DS Studio v16 software.

Hyperoside exhibited a highly favorable binding free energy (ΔG: −8.99 kcal/mol) against human pancreatic α-amylase (AAMY), indicating a strong binding affinity for the active site of this enzyme (Table 5). The compound formed a total of 12 H bonds with key residues in the active site, including Arg195 (1.88 Å, 3.00 Å), Asp197 (2.60 Å), Lys200 (2.26 Å), Glu233 (2.62 Å, 2.82 Å, 2.82 Å, 2.90 Å), Ile235 (1.84 Å, 3.27 Å), His299 (2.02 Å), and His305 (2.55 Å) (Table 5, Figure 7). These interactions of hyperoside reflect significant polar stabilization within the enzyme’s active site. The binding free energy of −8.99 kcal/mol for hyperoside was found close to that of the reference inhibitor montbretin A (−9.03 kcal/mol), indicating that hyperoside exhibits a binding affinity comparable to the native inhibitor.

**Figure 8:**
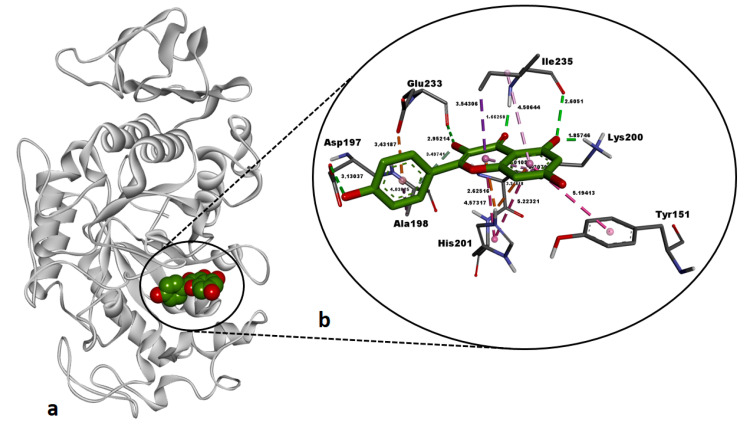
Post-docking top-ranked conformation of kaempferol in complex with the active site of human pancreatic alpha-amylase (AAMY). (**a**) 3D general view of the AAMY–kaempferol complex, with AAMY represented as a solid ribbon model and kaempferol in CPK mode; (**b**) zoomed-in interaction view of kaempferol within the active site of AAMY. Green dashed lines denote conventional hydrogen bonds, light pastel green dashed lines indicate carbon–hydrogen bonds, orange dashed lines signify electrostatic interactions, and dark and light purple dashed lines represent hydrophobic interactions. Non-bonded interaction distances (Å) are displayed in bold black. Images were rendered and prepared using DS Studio v16 software.

Kaempferol demonstrated a strong binding free energy (ΔG: −7.76 kcal/mol) against the active site of human pancreatic α-amylase (AAMY) (Table 5). The ligand formed four classical H bonds with Asp197 (3.13 Å), Lys200 (1.85 Å), Glu233 (2.95 Å), and Ile235 (1.66 Å, 2.60 Å), suggesting significant polar stabilization within the binding pocket (Table 5, Figure 8). In addition, kaempferol exhibited non-classical H bonds with Ala198 (3.49 Å) and His201 (2.62 Å, 3.24 Å). Extensive hydrophobic interactions were observed with residues such as Tyr151 (5.19 Å), Ala198 (4.03 Å), Lys200 (5.01 Å, 5.30 Å), His201 (4.57 Å, 5.22 Å), and Ile235 (3.54 Å, 4.50 Å), supporting the ligand’s stability in the active site. An electrostatic interaction with Glu233 (3.43 Å) further contributed to the stabilization of the ligand–enzyme complex (Table 5, Figure 8). The binding free energy of −7.76 kcal/mol for kaempferol, while weaker compared to the reference inhibitor montbretin A (−9.03 kcal/mol), represents a strong binding affinity, indicating an inhibitory potency within the active site of human AAMY enzyme.

Tropolone, the co-crystallized inhibitor of human tyrosinase-related protein 1 (TYRP1), exhibited a moderate binding affinity (ΔG: −5.08 kcal/mol) for the active site of TYRP1 (Table 5). Tropolone formed five classical H bonds with His192 (2.21 Å), His215 (2.01 Å), Gln390 (3.17 Å), Thr391 (3.20 Å), and Ser394 (1.94 Å), demonstrating polar interactions stabilizing the ligand–enzyme complex (Table 5, Supplementary Figure S4). Non-classical H bonds were formed with His192 (3.35 Å) and Thr391 (2.96 Å), further contributing to the binding stability. Hydrophobic contacts of tropolone were observed with His381 (3.67 Å), and an electrostatic π–cation interaction was noted with the same residue (4.03 Å) (Table 5, Supplementary Figure S4).

The docking interaction of chlorogenic acid against the active site of TYRP1 was not found to be energetically favorable (Table 5). Consequently, this ligand is considered an unlikely candidate as a potential bioactive component within the plant extract. The binding affinity value and intermolecular interactions of TYRP1 with 4-hydroxybenzoic acid is presented in Table 5 and Supplementary Figure S5 (see also Supplementary Materials).

**Figure 9:**
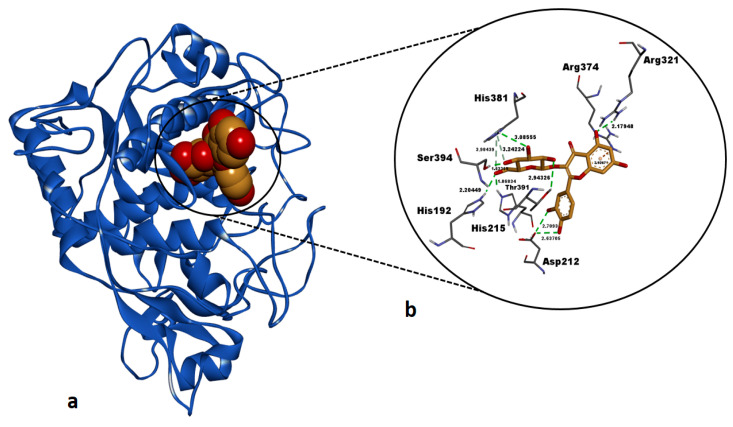
Post-docking top-ranked conformation of hyperoside in complex with the active site of human tyrosinase-related protein 1 (TYRP1). (**a**) 3D general view of the TYRP1–hyperoside complex, with TYRP1 represented as a solid ribbon model and hyperoside in CPK mode; (**b**) zoomed-in interaction view of hyperoside within the active site of TYRP1. Green dashed lines denote conventional hydrogen bonds, light pastel green dashed lines indicate pi-donor–hydrogen bonds, and orange dashed lines signify electrostatic interactions. Non-bonded interaction distances (Å) are displayed in bold black. Images were rendered and prepared using DS Studio v16 software.

Hyperoside showed a strong binding affinity (ΔG: −7.78 kcal/mol) against human tyrosinase-related protein 1 (TYRP1) active site (Table 5). The ligand formed seven classical H bonds with key residues: His192 (2.20 Å), Asp212 (2.63 Å, 2.70 Å), His215 (1.86 Å), Arg321 (2.17 Å), His381 (3.08 Å), Thr391 (2.94 Å), and Ser394 (1.62 Å) which underscore significant polar contributions to the ligand–receptor complex (Figure 9). Moreover, non-classical H bonds as well as an electrostatic interaction were formed with His381 (3.24 Å, 3.98 Å), and Arg374 (3.92 Å), respectively (Table 5, Figure 9). Hyperoside demonstrated a much stronger binding affinity (ΔG: −7.78 kcal/mol) compared to that of the inhibitor tropolone (ΔG: −5.08 kcal/mol), indicating that hyperoside could be more effective as an inhibitor of TYRP1.

**Figure 10:**
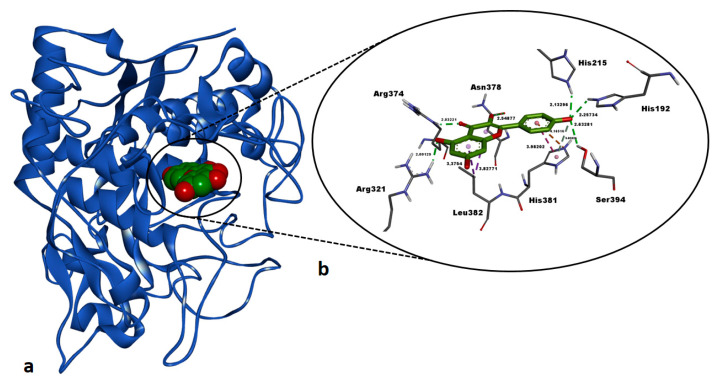
Post-docking top-ranked conformation of kaempferol in complex with the active site of human tyrosinase-related protein 1 (TYRP1). (**a**) 3D general view of the TYRP1– kaempferol complex, with TYRP1 represented as a solid ribbon model and kaempferol in CPK mode; (**b**) zoomed-in interaction view of kaempferol within the active site of TYRP1. Green dashed lines denote conventional hydrogen bonds, light pastel green dashed lines indicate pi-donor–hydrogen bonds, orange dashed lines signify electrostatic interactions, and dark and light purple dashed lines represent hydrophobic interactions. Non-bonded interaction distances (Å) are displayed in bold black. Images were rendered and prepared using DS Studio v16 software.

Kaempferol exhibited a strong binding affinity (ΔG: −7.62 kcal/mol) against the active site of human tyrosinase-related protein 1 (TYRP1) (Table 5). Kaempferol formed six classical H bonds with key residues: His192 (2.25 Å), His215 (2.13 Å), Arg321 (2.00 Å), Arg374 (2.03 Å), Asn378 (2.54 Å), and Ser394 (2.63 Å), forming many polar interactions (Figure 10). A non-classical H bond was also observed with His381 (3.83 Å), further supporting its binding mode. Hydrophobic interactions with His381 (3.98 Å) and Leu382 (3.37 Å, 3.82 Å) along with an electrostatic interaction with His381 (4.16 Å) (Table 5, Figure 10). Kaempferol exhibited a much stronger binding affinity (ΔG: −7.62 kcal/mol) than the co-crystallized inhibitor tropolone (ΔG: −5.08 kcal/mol), reflecting a significant inhibitory potency against TYRP1.

## 3. Discussion

The aerial part extract, which exhibited the highest TPC, TFC, and notable concentrations of diverse phenolic compounds, emerged as the most chemically rich extract, followed closely by the flower and leaf extracts. The root and stem extracts, despite their lower TPC and TFC, contributed uniquely to the overall phytochemical diversity, particularly through compounds like vanillin and syringic acid.

These observations suggest a positive correlation between the total phenolic and flavonoid contents and the concentrations of specific bioactive compounds, such as hyperoside, kaempferol, and luteolin 7-glucoside. Furthermore, the higher yields and phenolic content in the aerial part and flower extracts underline their prominence in future bioactivity-focused studies. Such findings emphasize the role of distinct plant parts in shaping the phytochemical and therapeutic potential of *A. melanophrurius*.

The chemical composition analysis of *A. melanophrurius* in this study reveals significant findings that add depth to the existing literature, particularly by highlighting the differential metabolite profiles between roots and aerial parts. While previous work primarily focused on root-derived triterpene glycosides, such as the eight cycloartane triterpene glycosides identified by Yürüker et al. [44], these compounds exhibited limited bioactivity apart from modest antibacterial and immunomodulatory effects. In contrast, the extraction yields and phytochemical analyses presented in the current study identified the aerial and flower extracts as the most chemically rich parts, with the highest total phenolic and flavonoid contents, along with significant concentrations of hyperoside, kaempferol, luteolin derivatives, and other bioactive compounds. These findings suggest that the aerial parts of *A. melanophrurius* hold greater potential for therapeutic applications due to their superior phytochemical profiles. Moreover, the observed inactivity of glycosides in broad bioassay systems further underscores the importance of targeting specific phenolic compounds found in other plant parts, such as leaves and flowers, for bioactive potential.

The notably higher concentrations of hyperoside in the leaves (573.21 µg/g) and aerial parts (242.19 µg/g) compared to the roots (not detected) indicate a strong correlation with the observed antioxidant and enzyme inhibitory activities. These findings further reinforce the link between the chemical richness of the aerial parts and their bioactivity potential. As indicated by prior studies on *Astragalus* species, phenolic compounds such as hyperoside, chlorogenic acid, and luteolin derivatives are known to contribute significantly to antioxidant and enzyme inhibitory effects [45,46]. The presence of these compounds in higher concentrations in aerial extracts supports the hypothesis that plant parts with elevated phenolic and flavonoid contents exhibit stronger bioactivity.

Furthermore, *A. melanophrurius* appears distinct from related species such as *A. tauricolus* and *A. trojanus*, which are rich in oleanane glycosides, cycloartane-type triterpene glycosides, and other saponins [47,48]. Unlike these species, which predominantly feature saponin profiles, our study highlights a broader phenolic spectrum in *A. melanophrurius*, particularly in the aerial parts. This chemotaxonomic diversity underscores the importance of investigating different plant parts, especially those rich in phenolic compounds, to uncover unique bioactive potentials.

The LC–ESI–MS/MS analysis provided critical insights into the distinct chemical profiles of various extracts. For instance, the aerial part extract exhibited higher concentrations of chlorogenic acid (28.77 µg/g), *p*-coumaric acid (26.19 µg/g), luteolin 7-glucoside (21.48 µg/g), and ferulic acid (21.04 µg/g), aligning with its superior total phenolic content (TPC) and total flavonoid content (TFC). These findings corroborate the earlier section’s conclusion that the aerial and flower extracts, which had the highest extract yields, are the most chemically and biologically active. Such phytochemical diversity emphasizes the need to focus on these parts in future pharmacological studies.

The leaf extract of *A. melanophrurius* emerged as the most potent antioxidant across multiple assays, likely due to its rich phenolic and flavonoid content. While other extracts displayed moderate activity, the RACI analysis corroborates the leaf extract’s preeminence. These findings emphasize the importance of integrating both assay-specific and holistic approaches when evaluating antioxidant capacities, highlighting the diverse mechanisms by which natural extracts mitigate oxidative stress.

The current study provides the first insight into the antioxidant activities of *A. melanophrurius*, filling a significant gap in the literature. While antioxidant properties of various Astragalus species have been reported, the results from this study highlight unique aspects of *A. melanophrurius* that distinguish it from its relatives. The outcomes of the assays applied here demonstrate diverse mechanisms of antioxidant activity, correlating well with specific phytochemical compositions.

The strong antioxidant activities observed in *A. melanophrurius* leaf and aerial part extracts are consistent with findings from closely related species. For instance, Shahrivari-Baviloliaei et al. [49] investigated the phenolic profile and antioxidant properties of *A. membranaceus* and found phenolic acids and flavonoids as key contributors. Notably, their study identified quercetin as a major active compound, aligning with the chemical composition of *A. melanophrurius* where quercetin was identified. These observations suggest that quercetin, along with other identified phenolic compounds, may play a pivotal role in the plant’s radical scavenging capacity by donating electrons to neutralize free radicals and interrupt oxidative chain reactions.

Similarly, Haşimi et al. [50] attributed the antioxidant activity of three endemic Anatolian *Astragalus* species to their phenolic and flavonoid contents, with chlorogenic acid and rutin playing significant roles. Chlorogenic acid is known to act as a hydrogen donor and metal chelator, thereby reducing the formation of reactive oxygen species (ROS). These findings support the hypothesis that the potent antioxidant effects of *A. melanophrurius* leaves in the DPPH and ABTS assays are largely driven by these compounds, which exhibit robust radical scavenging abilities by stabilizing unpaired electrons.

In the current study, the leaf extract exhibited superior DPPH radical scavenging activity compared to other plant parts, demonstrating its efficacy in neutralizing free radicals. This aligns with the results for *A. gymnolobus*, where Aydemir et al. [51] found the methanolic extract to exhibit comparable DPPH activity due to high phenolic content. The mechanism underlying this activity likely involves the interaction of phenolic hydroxyl groups with free radicals, leading to the formation of stable phenoxyl radicals, thereby halting oxidative damage.

The phosphomolybdenum assay results of *A. melanophrurius* root extract reflect significant reducing capacity, similar to *A. plumosus* var. *krugianus*, where Denizli et al. [52] reported strong antioxidant activity attributed to cycloartane glycosides. While glycosides were not prominently identified in *A. melanophrurius*, phenolics might serve as functional analogs contributing to the reducing power by transferring electrons to reduce oxidants. This reducing ability can disrupt oxidative stress pathways, which are critical in preventing lipid peroxidation and protein oxidation.

The study identified strong ferrous ion chelation by the flower extract, which is comparable to *A. abyssinicus*, where El Dib et al. [53] emphasized flavonoids as key iron chelators. Flavonoids, such as quercetin, can bind to metal ions through their hydroxyl and carbonyl groups, forming stable complexes that inhibit Fenton reactions and thereby limit the formation of highly reactive hydroxyl radicals. This highlights the potential role of flavonoids identified in *A. melanophrurius* in mitigating oxidative damage by reducing metal-catalyzed radical formation.

The novelty of this study lies in its comprehensive analysis of *A. melanophrurius* extracts across multiple antioxidant assays, coupled with RACI-based quantification for cross-mechanism comparisons. Unlike previous studies that focused on single extracts or specific assays, this study provides a robust comparative framework. The integration of phytochemical profiles with antioxidant activities enables deeper insights into structure–activity relationships, offering a holistic understanding of the species’ bioactivity. For instance, the study’s correlation analysis revealed that specific phenolic acids are likely responsible for the observed bioactivities through their electron-donating capacity, metal-chelating properties, and ability to stabilize reactive species.

The root extract consistently exhibited the highest inhibitory activity in both tyrosinase and α-amylase assays, suggesting it contains the most bioactive phytochemical profile among the tested extracts. Phenolic compounds and flavonoids likely contribute to this dual enzyme inhibition through mechanisms such as competitive binding to the enzyme’s active site and interference with substrate binding. These findings are consistent with previous studies on related *Astragalus* species, such as *A. membranaceus* and *A. plumosus*, where phenolics and flavonoids were identified as primary bioactives responsible for enzyme inhibitory activities [49,52].

The flower and stem extracts demonstrated comparatively lower activities in both assays, possibly due to lower concentrations of these key phytochemicals or the presence of compounds with lesser bioactivity. Notably, the aerial part extract performed better in α-amylase inhibition than expected, which may result from synergistic interactions between minor bioactives. The presence of diverse phenolic compounds can enhance enzyme inhibition through additive or synergistic effects, as different phenolic molecules may bind to distinct sites on the enzyme, thereby collectively improving inhibitory efficacy.

This study provides novel insights into the enzyme inhibitory activities of *A. melanophrurius*, marking its first evaluation in this context. The results highlight the root extract as a promising candidate for further investigation, particularly for its potential in skin depigmentation and diabetes management. Tyrosinase inhibition by the root extract is likely mediated by phenolic compounds that interact with copper ions at the enzyme’s active site, preventing the formation of melanin. Similarly, α-amylase inhibition can be attributed to phenolics’ ability to form hydrogen bonds with the enzyme, disrupting its catalytic function.

The significant α-amylase inhibitory activity demonstrated by the root extract of *A. melanophrurius* can be attributed to its unique phenolic composition, specifically the presence of major bioactive compounds such as *p*-coumaric acid, vanillic acid, vanillin, and syringic acid. Among these, *p*-coumaric acid has been highlighted as a potent inhibitor of α-amylase in recent studies. Huang et al. [54] explored its direct interaction with the enzyme’s active site and confirmed that it inhibits enzymatic function through competitive binding. This mechanistic insight aligns with the strong inhibitory activity observed in the root extract, suggesting that *p*-coumaric acid is a key contributor to its bioactivity.

In addition to *p*-coumaric acid, vanillin also demonstrates inhibitory effects on carbohydrate-metabolizing enzymes. Research by Liu et al. [55] supports its efficacy, particularly against α-glucosidase, and suggests potential cross-activity with α-amylase due to structural similarities with other inhibitory phenolics. Syringic acid, although less studied in the context of α-amylase inhibition, has shown moderate enzyme-inhibitory effects in various plant extracts, further emphasizing its contributory role. This combination of phenolics in the root extract likely functions synergistically, enhancing its overall efficacy by targeting multiple pathways in carbohydrate metabolism.

Corroborating evidence from studies on phenolic-rich plant extracts further strengthens this conclusion. For example, Tlili et al. [56] demonstrated significant α-amylase inhibition in *Amygdalus communis* hull extracts, which share a phenolic profile similar to that of *A. melanophrurius*. Similarly, Soria-Lopez et al. [57] reported strong enzyme-inhibitory effects in *Carica papaya* extracts, attributing these activities to their high phenolic content. These findings underline the critical role of phenolic compounds in enzyme inhibition and highlight the potential of such extracts in managing metabolic disorders like diabetes.

Overall, the remarkable α-amylase inhibitory activity of the root extract is driven primarily by *p*-coumaric acid, supported by the synergistic effects of vanillin and syringic acid. This unique composition positions the extract as a promising candidate for further development in anti-diabetic therapies, with the potential to act through multiple inhibitory mechanisms. Future studies should aim to isolate individual bioactives from the root extract and conduct molecular docking and kinetic analyses to better elucidate their precise mechanisms of action, thus paving the way for targeted therapeutic applications.

The PCA analysis reveals that hyperoside and phosphomolybdenum are the primary contributors to the variance in the antioxidant and enzyme inhibitor activities of the plant extracts. Their critical influence on Dim1 and Dim2 highlights their importance in explaining the chemical differences among plant parts. The strong alignment of leaves with hyperoside suggests that this phytochemical plays a significant role in the antioxidant activity of leaf extracts. Similarly, the proximity of root samples to phosphomolybdenum indicates that this compound heavily influences the antioxidant potential of root extracts.

The clustering patterns observed in the dendrogram provide additional insights into the chemical similarities and differences among plant parts. The grouping of stems and flowers in one cluster suggests a similar chemical composition, while the distinct separation of aerial parts underscores their unique phytochemical profile. The clustering of roots and leaves further supports the hypothesis that certain compounds, such as hyperoside and phosphomolybdenum, contribute significantly to the observed antioxidant activities.

These findings emphasize the importance of specific phytochemicals in determining the antioxidant and enzyme inhibitory activities of different plant organs. The alignment of certain plant parts with key compounds in the PCA biplots suggests targeted accumulation of bioactive compounds, which can be further explored for their potential therapeutic applications.

Our docking study revealed that hyperoside and kaempferol formed energetically strong interactions (Δ*G*: −8.99 kcal/mol and Δ*G*: −7.76 kcal/mol, respectively) with the catalytic residues of human pancreatic α-amylase, comparable to those of the co-crystallized inhibitor montbretin A (Table 5, Figure 7 and Figure 8). A recent study reported that hyperoside binds energetically favorably to α-amylase in docking simulations (Δ*G*: −8.5 kcal/mol) and forms H bonds with the catalytic residues Trp59, Gln63, Gly306, Asp197, Glu233, and Asp300 [58]. Consistent with this, our docking study revealed that hyperoside also forms H bonds with Asp197 and Glu233 and electrostatic interactions with Asp300 in AAMY active site (Table 5), aligning well with the literature. Additionally, another component of the *A. melanophrurius* Boiss. extract, kaempferol, has been shown in a separate study to form a highly favorable complex with human AAMY (Δ*G*: −8.1 kcal/mol) and to establish non-bonded interactions with residues in the enzyme’s binding pocket, including Trp58, Trp59, Tyr62, Gln63, His101, Leu162, Leu165, Arg195, Ala198, Glu233, Ile235, His299, and Asp300, which also contributed to experimental inhibition of this enzyme [59] (Table 5). Furthermore, previous studies have identified Asp197, Glu233, and Asp300 as key residues in the catalytic mechanism of AAMY [60,61]. These residue interactions are consistent with the findings observed in our study (Table 5) and suggest that both hyperoside and kaempferol hold promising potential as inhibitors of this enzyme. In our docking study, the second target receptor, human tyrosinase-related protein 1 (TYRP1), formed energetically favorable complexes with hyperoside and kaempferol, exhibiting binding free energies of −7.78 kcal/mol and −7.62 kcal/mol, respectively (Table 5, Figure 9 and Figure 10). Notably, the binding affinities of these two ligands were significantly more negative compared to the co-crystallized ligand, tropolone (Δ*G* = −5.08 kcal/mol) (Table 5), highlighting their stronger interaction with the target. To the best of our knowledge, no docking studies involving hyperoside and the TYRP1 enzyme have been reported in the literature. However, hyperoside demonstrated a binding affinity of −6.3 kcal/mol in docking experiments with tyrosinase isolated from *Agaricus bisporus* (mushroom tyrosinase), which can be considered indicative of a strong interaction [62]. In our study, the docking binding affinity of hyperoside against TYRP1 was significantly more favorable, suggesting the presence of additional amino acid side chains within the active site of human TYRP1 that positively influence hyperoside binding (Table 5). This is supported by the observation that hyperoside formed non-bonded interactions with critical residues—His192, His215, His381, Thr391, and Ser394—also involved in tropolone binding. Kaempferol, on the other hand, exhibited a binding energy of −5.58 kcal/mol against tyrosinase isolated from *Bacillus megaterium*, demonstrating its potential for interaction with tyrosinase enzymes [63]. In our study, kaempferol exhibited a significantly more favorable binding affinity against human TYRP1 (Δ*G* = −7.62 kcal/mol) (Table 5) compared to its reported interactions with prokaryotic tyrosinase [63]. This suggests that human TYRP1 is more sensitive to kaempferol and possess a greater inhibition potential. In summary, hyperoside and kaempferol may have undergone molecular evolution to establish optimal binding interactions with human TYRP1.

## 4. Materials and Methods

### 4.1. Plant Material

The plant *A. melanophrurius* was collected on June 12, 2022, from Isparta, Turkey, at an altitude of 1340 m (Senirkent-Suhut highway, 40–50 km roadside, coordinates: 38°13′22.86″ N, 30°41′31.29″ E). The species was identified by Lecturer Dr. Olcay Ceylan from the Department of Biology, Faculty of Science, Muğla Sıtkı Koçman University, and a voucher specimen has been deposited in the herbarium under the accession number OC.5011.

In this study, five different parts of the plant were used: leaves, stems, flowers, roots, and aerial portions. These parts were collected separately during the harvesting process and subsequently processed for extraction and analysis as described in the following sections. Each plant part was dried, ground into a fine powder, and stored at room temperature in a dry, dark environment prior to extraction.

### 4.2. Methanol Extraction

The dried plant material was divided into five distinct parts: leaves, stems, flowers, roots, and aerial portions. Each part was ground into a fine powder using a blender. From the ground plant material, 5 g portions were weighed and subjected to maceration in 100 mL of methanol for 24 h. This process was repeated once more, after which the extracts were pooled and concentrated under reduced pressure at approximately 40 °C using a rotary evaporator until all methanol was removed. After pooling the extracts, they were concentrated under reduced pressure using a vacuum system. The dried crude extracts were weighed, and the extraction yield (%) was calculated as follows:

$$Extraction\;yield\;\left(\%\right)=\frac{Weight\;of\;dried\;extract\;\left(g\right)}{Weight\;of\;dried\;plant\;material\;\left(g\right)}\times 100$$

The final concentrated extracts were obtained as semi-solid residues and stored at 4 °C until further use [64].

### 4.3. Determination of the Phenolic Compositions of the Extracts

While total phenolic and flavonoid analyses of the extracts were determined spectroscopically [65], their phytochemical contents were performed using a previously validated method [66], whose analytical characteristics are given in Tables S1 and S2 in the Supplementary Materials.

### 4.4. Biological Activity

Details of the antioxidant [65,67,68,69,70] and enzyme inhibitory activity [71] tests were given in Supplementary Materials.

### 4.5. Molecular Docking Experiments of Major Phytochemicals with Target Enzymes

To evaluate the potential enzyme inhibitory activities of major phytochemicals identified in the methanol (MeOH) extract of *A. melanophrurius,* molecular docking studies were conducted against two target enzymes: human pancreatic α-amylase (AAMY) and tyrosinase-related protein 1 (TYRP1). This computational approach provided insights into which phytochemical(s) might contribute to the observed inhibitory effects.

The crystal structures of AAMY complexed with montbretin A (PDB ID: 4W93, resolution: 1.35 Å) and TYRP1 complexed with tropolone (PDB ID: 5M8O, resolution: 2.50 Å) were retrieved from the RCSB Protein Data Bank (https://www.rcsb.org). The major phytochemicals—chlorogenic acid, 4-hydroxybenzoic acid, hyperoside, and kaempferol—were drawn using ChemOffice 19.1 software and saved in MOL format as ligands [72]. The co-crystallized inhibitors (montbretin A and tropolone) were extracted from the complexes and redocked into the active sites of the target proteins to validate the docking parameters. The obtained binding affinities (in kcal/mol) were used as reference values for comparison with the docking results of the identified phytochemicals.

### 4.6. Protein/Ligand Preparation and Molecular Docking

The crude structures of AAMY and TYRP1 were prepared for docking by removing peripheral water molecules and non-interacting ions using Discovery Studio Visualizer [73]. The geometries of chlorogenic acid, 4-hydroxybenzoic acid, hyperoside, and kaempferol were optimized using the General Amber Force Field (GAFF) in Avogadro software version 1.1.1 [74]. The optimized molecules were saved in MOL2 format and converted to PDBQT format using AutoDock Tools 1.5.7 [75].

### 4.7. Molecular Docking

Molecular docking simulations were performed using AutoDock 4.2.6. Polar hydrogen atoms were retained in the receptors and ligands, and charges were assigned using Kollman (for receptors) and Gasteiger (for ligands) charge models. The grid box dimensions for AAMY were set to 40 × 40 × 40 Å (center coordinates: x = −9.63, y = 4.34, z = −23.10), while for TYRP1, the dimensions were set to 17 × 17 × 17 Å (center coordinates: x = −13.34, y = 2.91, z = −25.38). A semi-flexible docking approach was employed, with receptors set as rigid and ligands as flexible.

The genetic algorithm (GA) parameters used for the docking simulations included a population size of 150, 5,000,000 evaluations, 27,000 generations, a mutation rate of 0.02, a crossover rate of 0.08, and 100 docking runs. The binding poses with the highest affinity (ΔG) and interactions similar to the co-crystallized inhibitors were selected for further analysis. The intermolecular interactions were visualized using DS Visualizer v16 software [73].

### 4.8. Statistical Analysis

The obtained results were presented as mean ± standard deviation (SD) based on three independent replicates (*n* = 3). Statistical significance was determined using ANOVA followed by Tukey’s post hoc test, with a significance threshold set at *p* < 0.05. The analyses were performed using SPSS version 26.0 and the Python programming 3.9.5 language.

To achieve a comprehensive evaluation of the antioxidant capacities of the extracts, Pearson correlation analysis, principal component analysis (PCA), and hierarchical cluster analysis (HCA) were performed. Given the different mechanisms of action underlying various antioxidant assays, direct comparisons of the results were not feasible. Therefore, the relative antioxidant capacity index (RACI) was calculated to standardize the results and facilitate meaningful comparisons across different assays.

RACI values were calculated by subtracting the mean value of each assay from the raw data and dividing the result by the standard deviation. Correlations between RACI values and individual antioxidant assays were also examined to identify the most influential assays in determining overall antioxidant capacity [76]. The detailed statistical analyses and additional figures are provided in the Supplementary Materials.

## 5. Conclusions

This study represents a comprehensive investigation into the chemical composition, antioxidant potential, and enzyme inhibitory activities of *A. melanophrurius* methanol extracts, revealing valuable insights into the bioactivity of this endemic plant. The detailed phytochemical analysis identified several bioactive compounds, including hyperoside and kaempferol, which appear to be the primary contributors to the observed biological activities. The leaf and root extracts, in particular, demonstrated noteworthy enzyme inhibitory potential, suggesting possible applications in managing diabetes and hyperpigmentation disorders.

However, several limitations should be addressed to fully elucidate the therapeutic potential of *A. melanophrurius*. While the phytochemical profile of the extracts was extensively characterized, the specific interactions and potential synergistic effects of individual compounds remain unclear. Fractionation and bioactivity-guided isolation approaches are essential to pinpoint the key constituents responsible for the observed bioactivities. Additionally, in vivo studies are critical to validate these findings under physiological conditions, particularly in the context of chronic diseases such as diabetes, where long-term efficacy and safety are paramount.

The moderate enzyme inhibitory activities observed compared to standard drugs suggest the need for structural optimization of the identified compounds to enhance their bioactivity. Future studies could benefit from integrating computational modeling with kinetic and thermodynamic analyses to better understand the molecular interactions between these compounds and their target enzymes.

Furthermore, expanding the research to investigate other pharmacological properties, such as anti-inflammatory, antimicrobial, and anticancer effects, could reveal additional therapeutic potential. The endemic nature of *A. melanophrurius* underscores the importance of preserving biodiversity while exploring its unique chemical profile for pharmaceutical applications.

Ultimately, this study lays a solid foundation for future research on *A. melanophrurius*, emphasizing the need for multidisciplinary approaches that combine advanced analytical techniques with biological and computational methods. Such integrative research will be instrumental in unlocking the full potential of this plant species for developing novel plant-based therapeutics and nutraceutical products.

## Figures and Tables

**Table 1:** Concentration (µg/g extract) of selected phenolic compounds in *A. melanophrurius* extracts.

Compounds	Flowers	Stems	Roots	Leaves	Aerial Parts
Hyperoside	14.65 ± 0.07 ^a^	16.68 ± 0.04 ^a^	nd	573.21 ± 3.80 ^d^	242.19 ± 3.45 ^c^
4-Hydroxybenzoic acid	202.74 ± 6.88 ^d^	18.89 ± 0.27 ^a^	38.12 ± 0.36 ^b^	34.36 ± 0.16 ^b^	80.56 ± 0.96 ^c^
Kaempferol	63.44 ± 5.03 ^d^	3.56 ± 0.90 ^ab^	nd	10.05 ± 0.17 ^b^	34.19 ± 1.60 ^c^
Chlorogenic acid	1.55 ± 0.02 ^a^	1.63 ± 0.01 ^a^	3.08 ± 0.23 ^a^	2.62 ± 0.02 ^a^	28.77 ± 1.20 ^b^
Vanillic acid	13.70 ± 1.43 ^a^	25.50 ± 2.36 ^bc^	30.81 ± 1.18 ^c^	21.04 ± 0.38 ^b^	27.41 ± 0.44 ^c^
*p*-Coumaric acid	17.11 ± 0.15 ^c^	15.20 ± 0.03 ^a^	15.01 ± 0.36 ^a^	23.34 ± 0.33 ^d^	26.19 ± 0.29 ^e^
Luteolin 7-glucoside	3.15 ± 0.04 ^b^	0.69 ± 0.02 ^a^	0.48 ± 0.10 ^a^	0.45 ± 0.09 ^a^	21.48 ± 0.39 ^c^
Ferulic acid	14.98 ± 0.39 ^b^	25.08 ± 0.27 ^d^	7.03 ± 0.13 ^a^	21.26 ± 0.25 ^c^	21.04 ± 0.16 ^c^
Verbascoside	1.15 ± 0.03 ^a^	1.39 ± 0.07 ^a^	2.44 ± 0.09 ^b^	1.65 ± 0.05 ^a^	12.23 ± 0.30 ^c^
Hesperidin	2.83 ± 0.01 ^a^	1.90 ± 0.06 ^a^	2.21 ± 0.11 ^a^	14.99 ± 1.20 ^c^	11.73 ± 0.38 ^b^
Protocatechuic acid	9.20 ± 0.12 ^b^	5.92 ± 0.20 ^a^	5.58 ± 0.23 ^a^	15.01 ± 0.17 ^c^	9.69 ± 0.23 ^b^
Vanillin	2.43 ± 0.03 ^a^	16.98 ± 0.49 ^d^	35.88 ± 0.32 ^e^	12.97 ± 0.58 ^c^	9.02 ± 0.25 ^b^
Gallic acid	2.83 ± 0.02 ^a^	3.55 ± 0.05 ^b^	2.86 ± 0.12 ^a^	8.65 ± 0.11 ^d^	5.92 ± 0.10 ^c^
Eriodictyol	0.32 ± 0.03 ^a^	1.04 ± 0.03 ^b^	3.14 ± 0.05 ^e^	2.31 ± 0.02 ^d^	1.61 ± 0.13 ^c^
Syringic acid	2.59 ± 0.21 ^a^	6.19 ± 0.11 ^b^	15.63 ± 0.74 ^c^	6.04 ± 0.22 ^b^	5.08 ± 0.20 ^b^
Rosmarinic acid	6.65 ± 0.09 ^b^	2.60 ± 0.14 ^a^	3.69 ± 0.05 ^a^	14.09 ± 1.16 ^c^	4.35 ± 0.21 ^a^
2,5-Dihydroxybenzoic acid	13.25 ± 0.74 ^b^	2.61 ± 0.10 ^a^	nd	2.72 ± 0.28 ^a^	3.08 ± 0.73 ^a^
Sinapic acid	3.15 ± 0.47 ^c^	2.34 ± 0.07 ^ab^	nd	1.70 ± 0.38 ^a^	2.95 ± 0.45 ^ab^
Quercetin	1.26 ± 0.02 ^a^	1.32 ± 0.04 ^a^	nd	2.16 ± 0.03 ^b^	2.03 ± 0.10 ^b^
Apigenin	2.09 ± 0.02 ^b^	nd	nd	0.86 ± 0.05 ^a^	1.94 ± 0.31 ^b^
Pinoresinol	nd	2.66 ± 0.20 ^b^	7.03 ± 0.05 ^c^	1.65 ± 0.24 ^a^	nd
3-Hydroxybenzoic acid	11.48 ± 0.02 ^b^	nd	nd	nd	3.54 ± 0.11 ^a^
Apigenin 7-glucoside	4.87 ± 0.18 ^a^	nd	nd	nd	6.36 ± 0.07 ^b^
(+)-Catechin	nd	nd	nd	nd	nd
Pyrocatechol	nd	nd	nd	nd	nd
(−)-Epicatechin	nd	nd	nd	nd	nd
Caffeic acid	nd	nd	nd	nd	nd
Taxifolin	nd	nd	nd	nd	nd
2-Hydroxycinnamic acid	nd	nd	nd	nd	nd
3,4-Dihydroxyphenylacetic acid	nd	nd	nd	nd	nd
Luteolin	nd	nd	nd	nd	nd

The results are presented as mean ± standard deviation (SD), based on three independent replicates (*n* = 3). The values indicated by the same superscripts within the same row are not different according to Tukey’s honestly significant difference post hoc test at 5% significance level. nd: not detected.

**Table 2:** Antioxidant activities of *A. melanophrurius* extracts.

Assays	Flowers	Stems	Roots	Leaves	Aerial Parts	Trolox	EDTA
DPPH radical (IC_50_: mg/mL)	47.08 ± 1.31 ^d^	35.32 ± 1.47 ^c^	>20 ^e^	16.55 ± 0.16 ^b^	>20 ^c^	0.26 ± 0.02 ^a^	-
ABTS radical cation (IC_50_: mg/mL)	6.19 ± 0.50 ^c^	9.32 ± 0.37 ^d^	9.83 ± 0.46 ^d^	4.58 ± 0.24 ^b^	6.50 ± 0.16 ^c^	0.27 ± 0.02 ^a^	-
Ferrous ion chelating (IC_50_: mg/mL)	1.04 ± 0.01 ^b^	1.16 ± 0.01 ^c^	1.24 ± 0.03 ^d^	1.16 ± 0.01 ^c^	1.09 ± 0.01 ^b^	-	0.034 ± 0.002 ^a^
Phosphomolybdenum (EC_50_: mg/mL)	3.23 ± 0.07 ^c^	2.62 ± 0.11 ^b^	1.40 ± 0.03 ^a^	2.95 ± 0.05 ^c^	2.98 ± 0.14 ^c^	1.14 ± 0.02 ^a^	-
CUPRAC reducing power (EC_50_: mg/mL)	5.12 ± 0.13 ^b^	11.29 ± 1.08 ^d^	7.35 ± 0.08 ^c^	4.12 ± 0.24 ^b^	5.03 ± 0.07 ^b^	0.32 ± 0.03 ^a^	-
FRAP reducing power (EC_50_: mg/mL)	6.54 ± 0.12 ^d^	6.92 ± 0.03 ^de^	7.22 ± 0.18 ^e^	3.07 ± 0.08 ^b^	3.91 ± 0.17 ^c^	0.11 ± 0.01 ^a^	-

TEs and EDTAEs mean trolox and ethylenediaminetetraacetic acid (disodium salt) equivalents, respectively. Values indicated by the same superscripts are not different from the honestly significant difference after Tukey’s hoc test at 5% significance level.

**Table 3:** Enzyme inhibition activity of *A. melanophrurius* extracts.

**Samples**	**Tyrosinase Inhibition** **(IC_50_: mg/mL)**	**α-Amylase Inhibition** **(IC_50_: mg/mL)**
Flowers	1.65 ± 0.05 ^c^	5.36 ± 0.17 ^c^
Stems	1.42 ± 0.12 ^bc^	6.62 ± 0.21 ^d^
Roots	1.34 ± 0.07 ^b^	2.99 ± 0.01 ^b^
Leaves	1.37 ± 0.01 ^b^	3.34 ± 0.01 ^b^
Aerial parts	1.47 ± 0.02 ^bc^	3.28 ± 0.01 ^b^
Kojic acid	0.37 ± 0.02 ^a^	-
Acarbose	-	1.19 ± 0.05 ^a^

ACEs and KAEs mean acarbose and kojic acid equivalents, respectively. Values indicated by the same superscripts are not different from the honestly significant difference after Tukey’s hoc test at 5% significance level.

**Table 4:** Correlations among phenolic compounds and assays.

	TAP	DPPH	ABTS	CUPRAC	FRAP	FICA
DPPH radical	−0.465					
ABTS radical cation	−0.614	0.822				
CUPRAC reducing power	−0.443	0.640	0.914			
FRAP reducing power	−0.454	0.922	0.848	0.794		
Ferrous ion chelating	−0.774	−0.043	0.345	0.378	0.077	
Total flavonoid	−0.734	0.391	0.799	0.850	0.536	0.793
Total phenolic	−0.197	−0.135	0.272	0.590	0.209	0.601
Chlorogenic acid	−0.226	0.058	0.063	0.256	0.366	0.320
4-Hydroxybenzoic acid	−0.395	−0.349	0.203	0.345	−0.216	0.820
Vanillic acid	0.668	−0.157	−0.557	−0.439	−0.088	−0.711
p-Coumaric acid	−0.515	0.654	0.674	0.740	0.871	0.333
Ferulic acid	−0.772	0.549	0.311	0.028	0.425	0.306
Luteolin 7-glucoside	−0.318	0.002	0.077	0.270	0.309	0.465
Hyperoside	−0.394	0.969	0.861	0.765	0.978	−0.037
Kaempferol	−0.577	−0.208	0.303	0.432	−0.046	0.926

Data show the Pearson correlation coefficients between the parameters. TAP: total antioxidant activity by phosphomolybdenum method. ABTS and DPPH: ABTS and DPPH radical scavenging activities, respectively. CUPRAC and FRAP: CUPRAC and FRAP reducing power potential; respectively. FICA: ferrous ion chelating activity.

**Table 5:** Molecular interactions of major phytochemicals and co-crystallized inhibitors with human pancreatic alpha-amylase (AAMY) and tyrosinase-related protein 1 (TYRP1), presenting docking binding affinity values (kcal/mol) and interaction patterns of ligands with amino acid residue side chains within the enzymesˈ active sites.

Compound	Molecular Weight (g/mol)	Receptor	Δ*G*_best_ (kcal/mol)	Classical H-Bond	Non-Classical H-Bond (Carbon-Hydrogen, pi-Donor)	Hydrophobic Contact (π–Sigma, Alkyl, π–Alkyl and π–π Stacked Interactions)	Electrostatic (pi-Cation, pi-Anion)	Miscellaneous (pi-Lone Pair)
Montbretin A (co-crystallized inhibitor)	1229.10	AAMY	−9.03	Ile148 (3.01 Å), Tyr151 (2.57 Å), Thr163 (1.97 Å, 2.21 Å, 3.19 Å), His201 (2.07 Å), Glu233 (2.56 Å), His299 (1.87 Å)	Tyr151 (3.49 Å), His305 (3.72 Å)	Leu162 (5.13 Å)	-	Tyr62 (2.83 Å)
Chlorogenic acid	354.310	AAMY	−3.80	**Tyr151** (2.99 Å), **Thr163** (1.97 Å, 2.24 Å), **His201** (1.86 Å), Ile235 (2.83 Å)	-	**Tyr151** (4.86 Å), Ile235 (4.99 Å)	-	-
4-hydroxybenzoic acid	138.12	AAMY	−4.12	Lys200 (1.69 Å), **Glu233** (2.81 Å), Ile235 (2.71 Å)	Ala198 (3.12 Å)	Lys200 (4.27 Å), **His201** (4.51 Å), Ile235 (3.73 Å)	**His201** (3.51 Å)	-
Hyperoside	464.40	AAMY	−8.99	Arg195 (1.88 Å, 3.00 Å), Asp197 (2.60 Å), Lys200 (2.26 Å), **Glu233** (2.62 Å, 2.82 Å, 2.82 Å, 2.90 Å), Ile235 (1.84 Å, 3.27 Å), **His299** (2.02 Å), **His305** (2.55 Å)	-	-	Asp300 (4.83 Å)	-
Kaempferol	286.24	AAMY	−7.76	Asp197 (3.13 Å), Lys200 (1.85 Å), **Glu233** (2.95 Å), Ile235 (1.66 Å, 2.60 Å)	Ala198 (3.49 Å), **His201** (2.62 Å, 3.24 Å)	**Tyr151** (5.19 Å), Ala198 (4.03 Å), Lys200 (5.01 Å, 5.30 Å), **His201** (4.57 Å, 5.22 Å), Ile235 (3.54 Å, 4.50 Å)	**Glu233** (3.43 Å)	-
Tropolone (co-crystallized inhibitor)	122.12	TYRP1	−5.08	His192 (2.21 Å), His215 (2.01 Å), Gln390 (3.17 Å), Thr391 (3.20 Å), Ser394 (1.94 Å)	His192 (3.35 Å), Thr391 (2.96 Å)	His381 (3.67 Å)	His381 (4.03 Å)	-
Chlorogenic acid	354.31	TYRP1	Unfavorable (highly positive binding energy)	-	-	-	-	-
4-hydroxybenzoic acid	138.12	TYRP1	−4.33	**His192** (1.86 Å), His224 (2.35 Å), His377 (1.75 Å), **Ser394** (3.06 Å), His404 (2.24 Å)	-	**His381** (3.52 Å)	**His381** (3.73 Å)	-
Hyperoside	464.40	TYRP1	−7.78	**His192** (2.20 Å), Asp212 (2.63 Å, 2.70 Å), **His215** (1.86 Å), Arg321 (2.17 Å), **His381** (3.08 Å), **Thr391** (2.94 Å), **Ser394** (1.62 Å)	**His381** (3.24 Å, 3.98 Å)	-	Arg374 (3.92 Å)	-
Kaempferol	286.24	TYRP1	−7.62	**His192** (2.25 Å), **His215** (2.13 Å), Arg321 (2.00 Å), Arg374 (2.03 Å), Asn378 (2.54 Å), **Ser394** (2.63 Å)	**His381** (3.83 Å)	**His381** (3.98 Å), Leu382 (3.37 Å, 3.82 Å)	**His381** (4.16 Å)	-

AAMY: Human Ppancreatic alpha-amylase in complex with montbretin A (PDB ID: 4W93). TYRP1: human tyrosinase related protein 1 in complex with tropolone (PDB ID: 5M8O). ΔG: best binding affinity value (kcal/mol) of the top-ranked docking pose. The amino acid residues highlighted in bold italics (Tyr151, Thr163, His201, Glu233, His299, and His305 for AAMY; His192, His215, His381, Thr391, and Ser394 for TYRP1) indicate that the major compounds exhibit binding interactions predominantly with the same residues as the native inhibitors, montbretin A and tropolone in docking simulations, demonstrating the efficacy of the docking conformational sampling algorithm.

## Data Availability

Data are contained within the article or Supplementary Materials.

## Supplementary Materials

The following supporting information can be downloaded at: https://www.mdpi.com/article/10.3390/ph18010103/s1, Figure S1: Visualization of the top-ranked docking interactions between AAMY and montbretin A (co-crystallized inhibitor); Figure S2: Visualization of the top-ranked docking interactions between AAMY and chlorogenic acid; Figure S3: Visualization of the top-ranked docking interactions between AAMY and 4-hydroxybenzoic acid; Figure S4: Visualization of the top-ranked docking interactions between TYRP1 and tropolone (co-crystallized inhibitor); Figure S5: Visualization of the top-ranked docking interactions between TYRP1 and 4-hydroxybenzoic acid; Table S1: ESI–MS/MS Parameters and analytical characteristics for the analysis of target analytes by MRM negative and positive ionization mode; Table S2: Calibration curves and sensitivity properties of the method.
