# Supplementary material for: Unveiling the Phytochemical Diversity and Bioactivity of Astragalus melanophrurius: A First Report Integrating Experimental and In Silico Approaches
Source: Pharmaceuticals (Basel). 2025 Jan 15;18(1):103. doi: 10.3390/ph18010103 (PMC11768182; doi:10.3390/ph18010103)
Supplement: Supplementary file 1 [file pharmaceuticals-18-00103-s001.zip › pharmaceuticals-3423107-supplementary.pdf]

SUPPLEMENTARY FILE

for

Unveiling the Phytochemical Diversity and Bioactivity of *Astragalus melanophrurius*: A First Report Integrating Experimental and In Silico Approaches

Figure S1 – Visualization of the top-ranked docking interactions between AAMY and montbretin A (co-crystallized inhibitor).

Figure S2 – Visualization of the top-ranked docking interactions between AAMY and chlorogenic acid.

Figure S3 – Visualization of the top-ranked docking interactions between AAMY and 4-hydroxybenzoic acid.

Figure S4 – Visualization of the top-ranked docking interactions between TYRP1 and tropolone (co-crystallized inhibitor).

Figure S5 – Visualization of the top-ranked docking interactions between TYRP1 and 4-hydroxybenzoic acid.

Results

Docking interactions between AAMY and chlorogenic acid

Docking interactions between AAMY and 4-hydroxybenzoic acid

Docking interactions between TYRP1 and 4-hydroxybenzoic acid

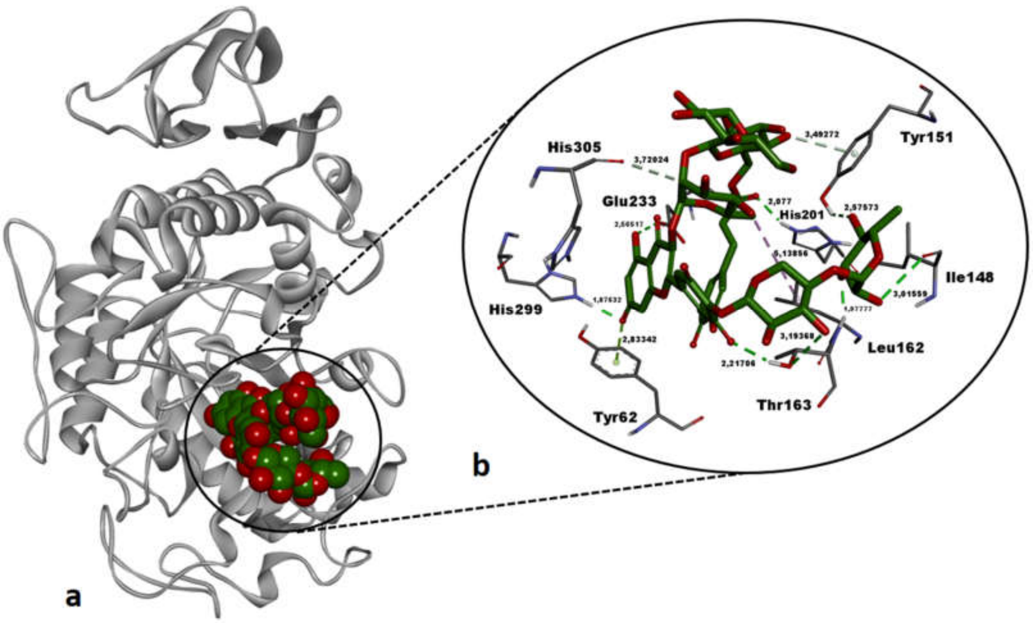

Figure S1 Post-docking top-ranked conformation of montbretin A (co-crystallized inhibitor) in complex with the active site of human pancreatic alpha-amylase (AAMY). (a) 3D general view of the AAMY–montbretin A complex, with AAMY represented as a solid ribbon model and montbretin A in CPK mode; (b) Zoomed-in interaction view of montbretin A within the active site of AAMY. Green dashed lines denote conventional hydrogen bonds, light pastel green dashed lines indicate carbon-hydrogen bonds and π-donor–hydrogen bonds, bright lime-colored dashed lines indicate π–lone pair interactions, and light purple dashed lines represent hydrophobic interactions. Non-bonded interaction distances (Å) are displayed in bold black. Images were rendered and prepared using DS Studio v16 software. CPK: Corey–Pauling–Koltun representation.

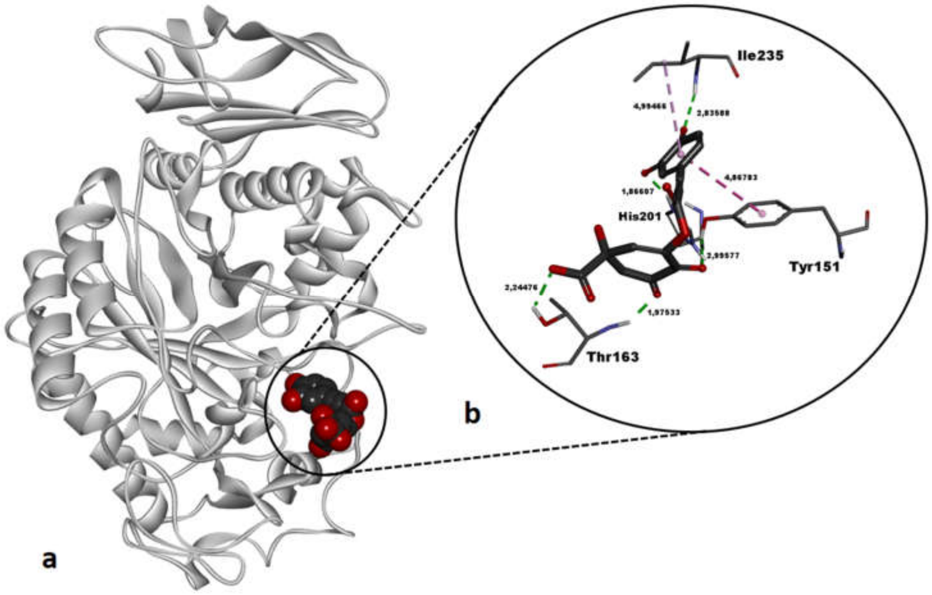

Figure S2 Post-docking top-ranked conformation of chlorogenic acid in complex with the active site of human pancreatic alpha-amylase (AAMY). (a) 3D general view of the AAMY– chlorogenic acid complex, with AAMY represented as a solid ribbon model and chlorogenic acid in CPK mode; (b) Zoomed-in interaction view of chlorogenic acid within the active site of AAMY. Green dashed lines denote conventional hydrogen bonds, and dark and light purple dashed lines represent hydrophobic interactions. Non-bonded interaction distances (Å) are displayed in bold black. Images were rendered and prepared using DS Studio v16 software. CPK: Corey–Pauling–Koltun representation.

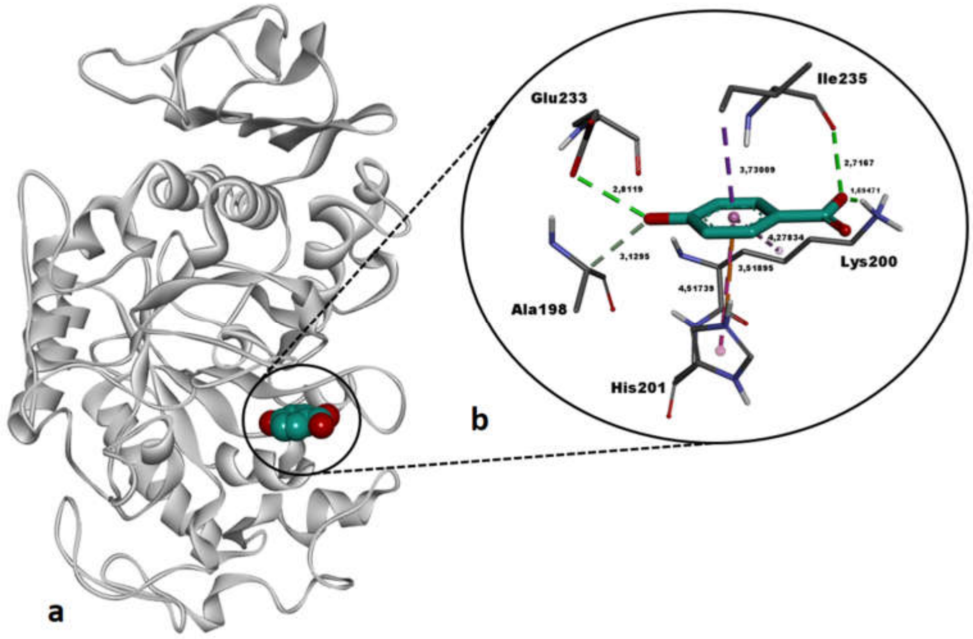

Figure S3 Post-docking top-ranked conformation of 4-hydroxybenzoic acid in complex with the active site of human pancreatic alpha-amylase (AAMY). (a) 3D general view of the AAMY–4-hydroxybenzoic acid complex, with AAMY represented as a solid ribbon model and 4-hydroxybenzoic acid in CPK mode; (b) Zoomed-in interaction view of 4-hydroxybenzoic acid within the active site of AAMY. Green dashed lines denote conventional hydrogen bonds, light pastel green dashed lines indicate carbon-hydrogen bonds, orange dashed lines signify electrostatic interactions, and dark and light purple dashed lines represent hydrophobic interactions. Non-bonded interaction distances (Å) are displayed in bold black. Images were rendered and prepared using DS Studio v16 software. CPK: Corey–Pauling–Koltun representation.

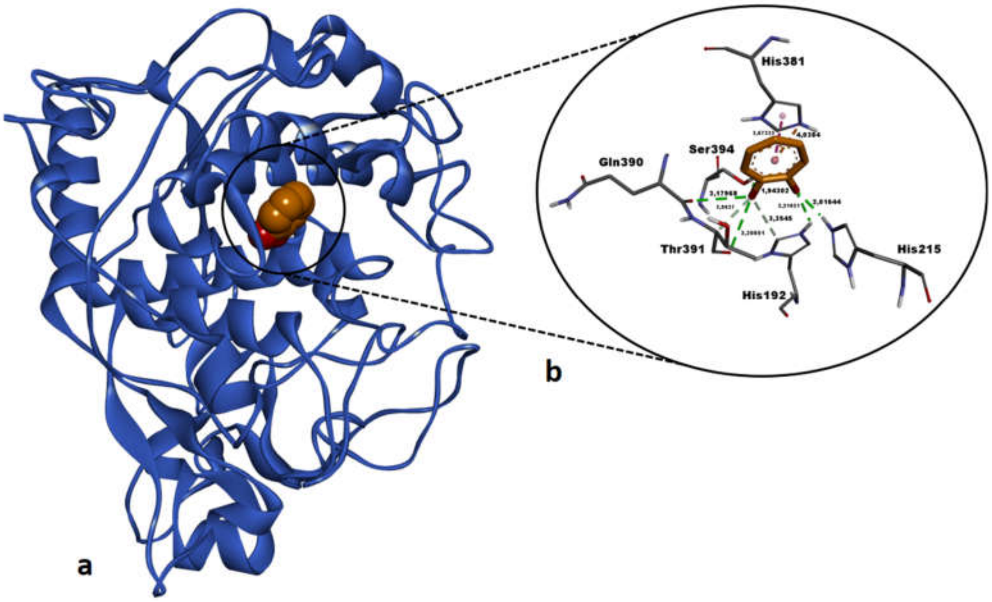

Figure S4 Post-docking top-ranked conformation of tropolone (co-crystallized inhibitor) in complex with the active site of human tyrosinase-related protein 1 (TYRP1). (a) 3D general view of the TYRP1–tropolone complex, with TYRP1 represented as a solid ribbon model and tropolone in CPK mode; (b) Zoomed-in interaction view of tropolone within the active site of TYRP1. Green dashed lines denote conventional hydrogen bonds, light pastel green dashed lines indicate carbon—hydrogen bonds, orange dashed lines signify electrostatic interactions, and dark purple dashed lines represent hydrophobic interactions. Non-bonded interaction distances (Å) are displayed in bold black. Images were rendered and prepared using DS Studio v16 software. CPK: Corey–Pauling–Koltun representation.

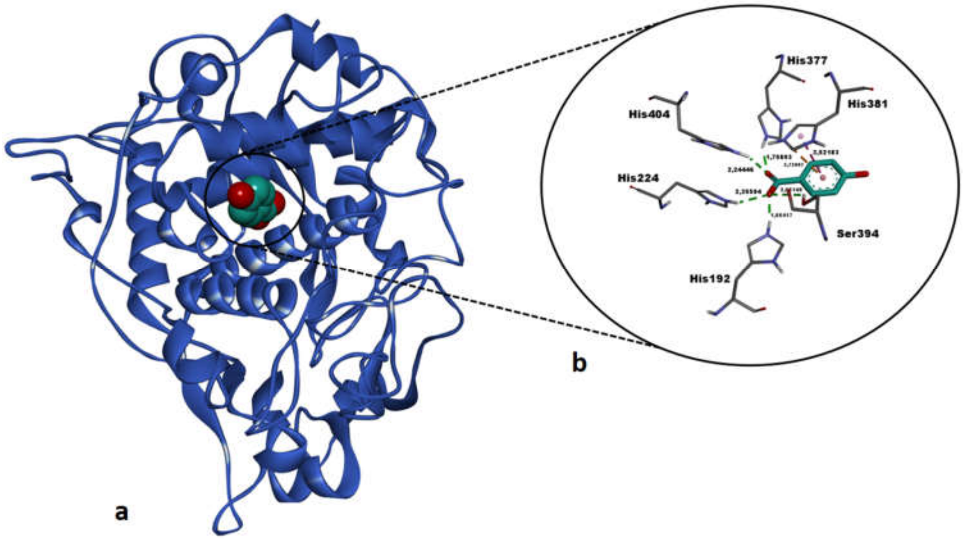

Figure S5 Post-docking top-ranked conformation of 4-hydroxybenzoic acid in complex with the active site of human tyrosinase-related protein 1 (TYRP1). (a) 3D general view of the TYRP1– 4-hydroxybenzoic acid complex, with TYRP1 represented as a solid ribbon model and 4-hydroxybenzoic acid in CPK mode; (b) Zoomed-in interaction view of 4-hydroxybenzoic acid within the active site of TYRP1. Green dashed lines denote conventional hydrogen bonds, orange dashed lines signify electrostatic interactions, and dark purple dashed lines represent hydrophobic interactions. Non-bonded interaction distances (Å) are displayed in bold black. Images were rendered and prepared using DS Studio v16 software. CPK: Corey–Pauling–Koltun representation.

Results

Docking interactions between AAMY and chlorogenic acid

Chlorogenic acid exhibited a weak binding interaction (Δ*G*: -3.80 kcal/mol) against the active site of human pancreatic alpha-amylase (AAMY). The compound formed a total of 5 hydrogen bonds with key residues, including Tyr151 (2.99 Å), Thr163 (1.97 Å, 2.24 Å), His201 (1.86 Å), and Ile235 (2.83 Å). In addition, chlorogenic acid displayed hydrophobic contacts with Tyr151 (4.86 Å) and Ile235 (4.99 Å), suggesting complementary interactions within the active site.

Docking interactions between AAMY and 4-hydroxybenzoic acid

4-hydroxybenzoic acid, demonstrated a weak binding interaction (Δ*G*: -4.12 kcal/mol) against human pancreatic alpha-amylase (AAMY) within the enzyme's active site. The compound formed three classical hydrogen bonds with residues Lys200 (1.69 Å), Glu233 (2.81 Å), and Ile235 (2.71 Å). In addition, 4-hydroxybenzoic acid engaged in non-classical hydrogen bonding with Ala198 (3.12 Å) and hydrophobic contacts with Lys200 (4.27 Å), His201 (4.51 Å), and Ile235 (3.73 Å). Furthermore, the ligand exhibited electrostatic interactions *via* His201 (3.51 Å).

Docking interactions between TYRP1 and 4-hydroxybenzoic acid

4-hydroxybenzoic acid exhibited a relatively weak binding affinity (Δ*G*: -4.33 kcal/mol) against human TYRP1. The ligand formed five classical hydrogen bonds with His192 (1.86 Å), His224 (2.35 Å), His377 (1.75 Å), Ser394 (3.06 Å), and His404 (2.24 Å), demonstrating key polar interactions contributing to the binding. Hydrophobic interactions were observed with His381 (3.52 Å), and an electrostatic interaction with His381 (3.73 Å), the same residue, was also noted.

Phytochemical analysis

A simple, rapid, reproducible, and sensitive method, which was previously developed and validated, was used for the simultaneous determination of 31 phenolic compounds using LC–ESI–MS/MS. An Agilent Technologies 1260 Infinity liquid chromatography system hyphenated to a 6420 Triple Quad mass spectrometer was used for quantitative analyses. Chromatographic separation was carried out on a Poroshell 120 EC-C18 (100 mm × 4.6 mm I.D., 2.7 μm) column. The mobile phase configuration (0.1% formic acid/methanol) was selected on the base of the better chromatographic resolution of isomeric compounds. On the other hand, the selected mobile phase configuration also provided higher sensitivity for many of the phenolic compounds. As a result, the mobile phase was made up from solvent A (0.1%, *v/v* formic acid solution) and solvent B (methanol). The gradient profile was set as follows: 0.00 min 2% B eluent, 3.00 min 2% B eluent, 6.00 min 25% B eluent, 10.00 min 50% B eluent, 14.00 min 95% B eluent, 17.00 min 95% B and 17.50 min 2% B eluent. The column temperature was maintained at 25°C. The flow rate was 0.4 mL min^{-1} and the injection volume was 2.0 μL. The tandem mass spectrometer was interfaced to the LC system via an ESI source. The electrospray source of the MS was operated in negative and positive multiple reaction monitoring (MRM) mode and the interface conditions were as follows: capillary voltage of −3.5 kV, gas temperature of 300°C and gas flow of 11 L min^{-1}. The nebulizer pressure was 40 psi.

In negative and positive multiple reaction monitoring (MRM) mode, the peaks of the analytes were identified by comparing the retention time, together with the monitoring ions pairs in an authentic standard solution.

Table S1. ESI–MS/MS Parameters and analytical characteristics for the Analysis of Target Analytes by MRM Negative and Positive Ionization Mode

Target compounds	Rt (min)	Precursor ion	MRM1 (CE, V)	MRM2 (CE, V)
Compounds analyzed by NI mode				
Gallic acid	8.891	168.9 [M − H]−	125.0 (10)	–
Protocatechuic acid	10.818	152.9 [M − H]−	108.9 (12)	–

3,4-Dihydroxyphenylacetic acid	11.224	167.0 [M − H]−	123.0 (2)	–
(+)-Catechin	11.369	289.0 [M − H]−	245.0 (6)	202.9 (12)
Pyrocatechol	11.506	109.0 [M − H]−	90.6 (18)	52.9 (16)
2,5-Dihydroxybenzoic acid	12.412	152.9 [M − H]−	109.0 (10)	–
4-Hydroxybenzoic acid	12.439	136.9 [M − H]−	93.1 (14)	–
Caffeic acid	12.841	179.0 [M − H]−	135.0 (12)	–
Vanillic acid	12.843	166.9 [M − H]−	151.8 (10)	122.6 (6)
Syringic acid	12.963	196.9 [M − H]−	181.9 (8)	152.8 (6)
3-Hydroxybenzoic acid	13.259	137.0 [M − H]−	93.0 (6)	–
Vanillin	13.397	151.0 [M − H]−	136.0 (10)	–
Verbascoside	13.589	623.0 [M − H]−	461.0 (26)	160.8 (36)
Taxifolin	13.909	303.0 [M − H]−	285.1 (2)	125.0 (14)
Sinapic acid	13.992	222.9 [M − H]−	207.9 (6)	163.8 (6)
p-Coumaric acid	14.022	162.9 [M − H]−	119.0 (12)	–
Ferulic acid	14.120	193.0 [M − H]−	177.8 (8)	134.0 (12)
Luteolin 7-glucoside	14.266	447.1 [M − H]−	285.0 (24)	–
Rosmarinic acid	14.600	359.0 [M − H]−	196.9 (10)	160.9 (10)
2-Hydroxycinnamic acid	15.031	162.9 [M − H]−	119.1 (10)	–
Pinoresinol	15.118	357.0 [M − H]−	151.0 (12)	135.7 (34)
Eriodictyol	15.247	287.0 [M − H]−	151.0 (4)	134.9 (22)
Quercetin	15.668	301.0 [M − H]−	178.6 (10)	151.0 (16)
Kaempferol	16.236	285.0 [M − H]−	242.8 (16)	229.1 (18)
Compounds analyzed by PI mode				
Chlorogenic acid	11.802	355.0 [M + H]+	163.0 (10)	–
(−)-Epicatechin	12.458	291.0 [M + H]+	139.1 (12)	122.9 (36)
Hesperidin	14.412	611.1 [M + H]+	449.2 (4)	303.0 (20)
Hyperoside	14.506	465.1 [M + H]+	303.1 (8)	–
Apigenin 7-glucoside	14.781	433.1 [M + H]+	271.0 (18)	–
Luteolin	15.923	287.0 [M + H]+	153.1 (34)	135.1 (36)
Apigenin	16.382	271.0 [M + H]+	153.0 (34)	119.1 (36)

R_t, retention time; NI, negative ion; and PI, positive ion.

Table S2. Calibration curves and sensitivity properties of the method

Compounds	Linearity and sensitivity characteristics				
	Range (μg/L)	Linear equation	R^2	LOD (μg/L)	LOQ (μg/L)
Gallic acid	5–500	y = 4.82x − 26.48	0.9988	1.46	4.88
Protocatechuic acid	2.5–500	y = 5.65x − 9.99	0.9990	1.17	3.88
3,4-Dihydroxyphenylacetic acid	5–500	y = 5.13x − 12.39	0.9990	1.35	4.51
(+)-Catechin	10–500	y = 1.45x + 1.95	0.9974	3.96	13.20
Pyrocatechol	25–400	y = 0.11x − 0.52	0.9916	9.62	32.08
Chlorogenic acid	1–500	y = 12.14x + 32.34	0.9995	0.55	1.82
2,5-Dihydroxybenzoic acid	5–500	y = 3.79x − 14.12	0.9980	2.12	7.08
4-Hydroxybenzoic acid	5–500	y = 7.62x + 22.79	0.9996	1.72	5.72
(−)-Epicatechin	5–500	y = 9.11x − 9.99	0.9971	1.85	6.18
Caffeic acid	5–500	y = 11.09x + 16.73	0.9997	3.15	10.50
Vanillic acid	10–500	y = 0.49x − 1.61	0.9968	2.56	8.54
Syringic acid	10–500	y = 0.74x − 1.54	0.9975	3.75	12.50
3-Hydroxybenzoic acid	5–500	y = 3.69x − 12.29	0.9991	1.86	6.20
Vanillin	50–500	y = 2.02x + 135.49	0.9926	15.23	50.77
Verbascoside	2.5–500	y = 8.59x − 28.05	0.9988	0.82	2.75
Taxifolin	5–500	y = 12.32x + 9.98	0.9993	1.82	6.05
Sinapic acid	5–500	y = 2.09x − 6.79	0.9974	2.64	8.78
p-Coumaric acid	5–500	y = 17.51x + 53.73	0.9997	1.93	6.44
Ferulic acid	5–500	y = 3.32x − 4.30	0.9992	1.43	4.76
Luteolin 7-glucoside	1–500	y = 45.25x + 156.48	0.9996	0.45	1.51
Hesperidin	5–500	y = 5.98x + 0.42	0.9993	1.73	5.77
Hyperoside	2.5–500	y = 16.32x − 1.26	0.9998	0.99	3.31
Rosmarinic acid	1–500	y = 9.82x − 17.98	0.9989	0.57	1.89
Apigenin 7-glucoside	1–500	y = 21.33x − 31.69	0.9983	0.41	1.35
2-Hydroxycinnamic acid	1–500	y = 16.72x − 26.94	0.9996	0.61	2.03
Pinoresinol	10–500	y = 0.80x − 2.69	0.9966	3.94	13.12
Eriodictyol	2.5–500	y = 14.24x − 0.50	0.9998	0.80	2.68
Quercetin	5–500	y = 14.68x − 18.25	0.9997	1.23	4.10
Luteolin	5–500	y = 8.96x + 26.80	0.9992	1.34	4.46
Kaempferol	10–500	y = 0.82x − 3.06	0.9959	3.30	10.99
Apigenin	2.5–500	y = 11.29x + 38.05	0.9987	0.96	3.20

LOD and LOQ: limit of detection and limit of quantification, respectively.

Biological activity

For total phenolic content, sample solution (0.25 mL) was mixed with diluted Folin-Ciocalteu reagent (1 mL, 1:9) and shaken vigorously. After 3 min, Na_2CO_3 solution (0.75 mL, 1%) was added and the sample absorbance was read at 760 nm after 2 h incubation at room temperature. Total phenolic content was expressed as equivalents of gallic acid.

For total flavonoid content, sample solution (1 mL) was mixed with the same volume of aluminium trichloride (2%) in methanol. Similarly, a blank was prepared by adding sample solution (1 mL) to methanol (1 mL) without $AlCl_3$. The sample and blank absorbance were read at 415 nm after 10 min incubation at room temperature. Absorbance of the blank was subtracted from that of the sample. Total flavonoid content was expressed as equivalents of rutin.

Total antioxidant activity of the samples was evaluated by phosphomolybdenum method. Sample solution (0.2 mL) was combined with 2 mL of reagent solution (0.6 M sulfuric acid, 28 mM sodium phosphate and 4 mM ammonium molybdate). The sample absorbance was read at 695 nm after 90 min incubation at 95°C.

For 1,1-diphenyl-2-picrylhydrazyl (DPPH) radical scavenging activity, sample solution (1 mL) was added to a 4 mL of 0.004% methanol solution of DPPH. Sample absorbance was read at 517 nm after 30 min incubation at room temperature in dark.

For ABTS cation radical scavenging activity, briefly, $ABTS^{\cdot+}$ radical cation was produced directly by reacting 7 mM ABTS solution with 2.45 mM potassium persulfate and allowing the mixture to stand for 12-16 h in dark at the room temperature. Prior to beginning the assay, ABTS solution was diluted with methanol to obtain an absorbance of 0.700 ± 0.02 at 734 nm. Sample solution (1 mL) was added to ABTS solution (2 mL) and mixed. Sample absorbance was read at 734 nm after 7 min incubation at room temperature.

For metal chelating activity on ferrous ions, briefly, sample solution (2 mL) was added to $FeCl_2$ solution (0.05 mL, 2 mM). The reaction was initiated by the addition of 5 mM ferrozine (0.2 mL). Similarly, a blank was prepared by adding sample solution (2 mL) to $FeCl_2$ solution (0.05 mL, 2 mM) and water (0.2 mL) without ferrozine. Then, the sample and blank absorbance were read at 562 nm after 10 min incubation at room temperature.

For cupric ion reducing activity (CUPRAC), sample solution (0.5 mL) was added to a premixed reaction mixture containing $CuCl_2$ (1 mL, 10 mM), neocuproine (1 mL, 7.5 mM) and NH_4Ac buffer (1 mL, 1 M, pH 7.0). Similarly, a blank was prepared by adding sample solution (0.5 mL) to a premixed reaction

mixture (3 mL) without $CuCl_2$. Then, the sample and blank absorbance were read at 450 nm after 30 min incubation at room temperature.

For ferric reducing antioxidant power (FRAP), sample solution (0.1 mL) was added to a premixed FRAP reagent (2 mL) containing acetate buffer (0.3 M, pH 3.6), 2,4,6-tris(2-pyridyl)-s-triazine (TPTZ) (10 mM) in 40 mM HCl and ferric chloride (20 mM) in a ratio of 10:1:1 (v/v/v). Then, the sample absorbance was read at 593 nm after 30 min incubation at room temperature.

Inhibitory activity on α-amylase was performed using Caraway-Somogyi iodine/potassium iodide (IKI) method. Sample solution (25 μL) was mixed with α-amylase solution (50 μL) in phosphate buffer (pH 6.9 with 6 mM sodium chloride) in a 96-well micro plate and incubated for 10 min at 37°C. After pre-incubation, the reaction was initiated by the addition of starch solution (50 μL, 0.05%). Similarly, a blank was prepared by adding sample solution to all reaction reagents without enzyme solution (α-amylase). The reaction mixture was incubated 10 min at 37°C. The reaction was then stopped with the addition of HCl (25 μL, 1 M). This was followed by the addition of iodine-potassium iodide solution (100 μL). The sample and blank absorbance were read at 630 nm. Absorbance of the blank was subtracted from that of the sample.

Tyrosinase inhibitory activity was measured using a modified dopachrome method with L-DOPA as substrate. Sample solution (25 μL) was mixed with tyrosinase solution (40 μl) and phosphate buffer (100 μl, pH 6.8) in a 96-well microplate and incubated for 15 min at 25°C. The reaction was then initiated with the addition of L-DOPA (40 μl). Similarly, a blank was prepared by adding sample solution to all reaction reagents without enzyme (tyrosinase) solution. The sample and blank absorbance were read at 492 nm after 10 min incubation at 25°C.

The sample concentration, which decreases the initial concentration by 50% for enzyme inhibition, radical scavenging and metal chelation tests, was defined as IC_{50}, while the EC_{50} values were calculated as sample concentration providing 0.500 absorbance for reducing power and phosphomolybdenum assays. The biological activities of the extracts were expressed as mg standard equivalent/g extract and compared with those of the standards, including trolox, ethylenediaminetetraacetic acid (disodium salt) (EDTA), galanthamine, kojic acid, and acarbose, used as positive controls.
